# Scheme for generation of three-photon entangled *W* state assisted by cross-Kerr nonlinearity and quantum dot

**DOI:** 10.1038/s41598-019-46231-7

**Published:** 2019-07-12

**Authors:** Jino Heo, Changho Hong, Seong-Gon Choi, Jong-Phil Hong

**Affiliations:** 1 College of Electrical and Computer Engineering, Chungbuk National University, Chungdae-ro 1, Seowon-Gu, Cheongju Republic of Korea; 2 Base Technology Division, National Security Research Institute, P.O. Box 1, Yuseong, Daejeon 34188 Republic of Korea

**Keywords:** Quantum information, Quantum optics, Quantum information, Quantum optics

## Abstract

We represent an optical scheme using cross-Kerr nonlinearities (XKNLs) and quantum dot (QD) within a single-sided optical cavity (QD-cavity system) to generate three-photon entangled *W* state containing entanglement against loss of one photon of them. To generate *W* state (three-photon) with robust entanglement against loss of one photon, we utilize effects of optical nonlinearities in XKNLs (as quantum controlled operations) and QD-cavity system (as a parity operation) with linearly optical devices. In our scheme, the nonlinear (XKNL) gate consists of weak XKNLs, quantum bus beams, and photon-number-resolving measurement to realize controlled-unitary gate between two photons while another nonlinear (QD) gate employs interactions of photons and an electron of QD confined within a single-sided optical cavity for implementation of parity gate. Subsequently, for the efficiency and experimental feasibility of our scheme generating *W* state, we analyze the immunity of the controlled-unitary gate using XKNLs against decoherence effect and reliable performance of parity gate using QD-cavity system.

## Introduction

Quantum entanglement due to features such as Bell state, Greenberger-Horne-Zeilinger (GHZ) state, and so on different from classical physics plays a significant role in quantum information processing (QIP) schemes such as quantum communications^1–8^, quantum computations^9–14^, quantum entanglement^15–20^, and quantum channel^21–25^. However, in the case of multi-qubit entangled state, it is difficult to maintain correlation of entanglement between all qubits for QIP scheme under the loss of qubit. For example, if one qubit of three qubits in GHZ state is traced out (or loss), the remaining two qubits cannot be correlated with each other.

From this point of view, to contain entanglement against loss of one qubit eliminated (or traced out) in *W* state^18,26–29^, which can be classified to three-qubit (non-maximally) entangled states as $|W\rangle \equiv (|001\rangle +|010\rangle +\sqrt{2}|100\rangle )/2$ (perfect *W* state), correlation of two qubits can be preserved. Therefore, various QIP schemes, quantum communications^28,30–33^, computing^34–36^, and quantum channels^37–39^ have exploited the entangled *W* states as essential resource for applications in QIP.

To experimentally implement diverse QIP schemes, cross-Kerr nonlinearities (XKNLs)^12,14,25,40–46^ and quantum dots (QDs) inside micro-cavities (QD-cavity systems)^4,6,7,23,47–54^ have been extensively studied to design multi-qubit gate for quantum controlled operations. Furthermore, decoherence effect which is induced by photon loss and dephasing^41,42,46,55–57^ in XKNLs can be decreased by utilizing photon-number-resolving (PNR) measurement and quantum bus (qubus) beams or displacement operator when increasing the amplitude of coherent state^41,42,46^. Also, in QDs within cavities (QD-cavity systems) during interaction between photons and QDs, quantum information (electron spin) can be stored for a long-term by long electron spin coherence time (${{\rm{T}}}_{{\rm{2}}}^{{\rm{e}}} \sim {\rm{\mu }}s$)^58,59^ for a limited spin relaxation time (${{\rm{T}}}_{1}^{{\rm{e}}}\sim m{\rm{s}}$)^60–62^ in order to reliable performance for designed QIP schemes.

In this paper, we propose an optical scheme via XKNLs (for controlled operations) and a QD-cavity system (for parity operation) to generate three-photon *W* state having the robust entanglement against loss of one photon (traced out). To generate three-photon *W* state, our scheme consists of two controlled-unitary [controlled-Hadamard and controlled-NOT (CNOT)] gates employing weak XKNLs, qubus (probe) beams, and PNR measurements^12,14,25,46^, and a parity gate using interaction between photons and an excess electron of QD confined in a single-sided cavity^4,6,7,23,47–54^ and linearly optical devices [circular polarizing beam splitters (CPBSs), beam splitters (BSs), and single qubit gates]. For nonlinearly optical gates (using XKNLs and QD-cavity system), we will analyze the influence to reduce fidelity of quantum state and reliable performance by decoherence effect in XKNLs and by vacuum noise and sideband leakage and absorption of optical cavity in QD-cavity system. Consequently, our scheme can be feasible and realized for the generation of three-photon *W* state as $|W\rangle \equiv ({|RRL\rangle }_{{\rm{ABC}}}+{|RLR\rangle }_{{\rm{ABC}}}+\sqrt{2}{|LRR\rangle }_{{\rm{ABC}}})/2$ through our analysis of efficiency and performance of nonlinearly optical gates (XKNLs: controlled-unitary gates and QD-cavity system: parity gate).

## Basic Concepts of Interactions in XKNLs and QD-Cavity System

### Interaction of XKNL in Kerr medium

**Figure 1:**
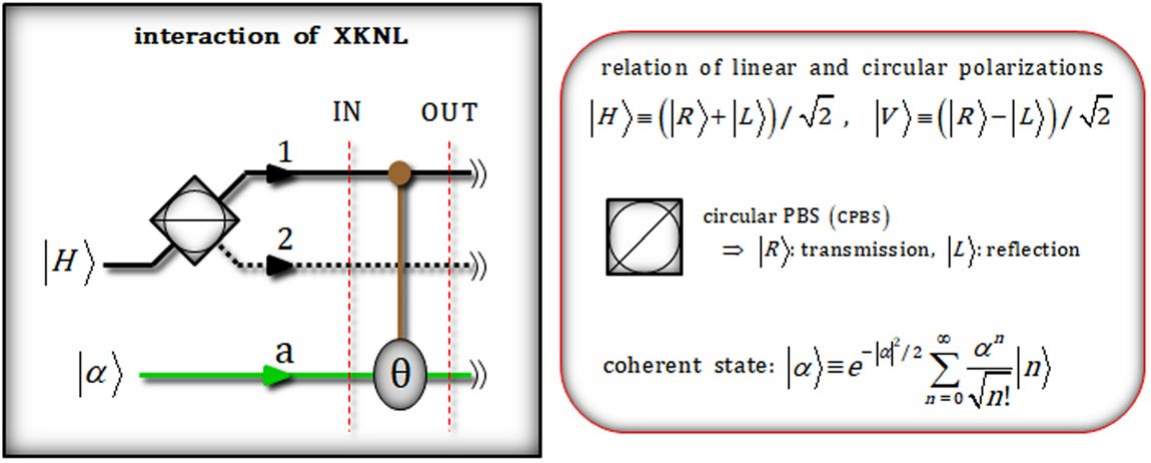
Plot schematically represents the interaction of XKNL between a photon and probe beam (coherent state). After this interaction, the conditional phase shift, θ, in the phase space of coherent state is (or not) induced by Kerr effect due to polarization ($|R\rangle$ or $|L\rangle$) of photon. Here, a photon plays the role of control qubit (signal) which can perform conditional phase shift to target system (probe beam: coherent state).

We introduce XKNL’s Hamiltonian as *H*_*Kerr*_ = ℏ*χN*_1_*N*_2_, where *N*_*i*_ and *χ* are photon number operator and strength of nonlinearity in Kerr medium. Figure 1 shows the interaction of XKNL between a photon (control) and probe beam (coherent state: target) to induce phase shift in Kerr medium. To describe the interaction of XKNL, we assume the input system of a photon, having linear polarization ($|H\rangle$: horizontal), and coherent state $|\alpha \rangle$. After CPBS splits the polarization of photon with regarding to circular polarizations ($|R\rangle$: right and $|L\rangle$: left), the input system (step IN) is transformed as

1 $$|H\rangle \otimes |\alpha {\rangle }^{a}\mathop{\to }\limits^{{\rm{C}}{\rm{B}}{\rm{P}}{\rm{S}}}\frac{1}{\sqrt{2}}(|R{\rangle }^{1}+|L{\rangle }^{2})\otimes |\alpha {\rangle }^{a}.\,[{\rm{I}}{\rm{N}}]$$

The operation (U_*Kerr*_: conditional phase shift) between a photon (control) and probe beam (target) by XKNL is expressed as

2 $${{\rm{U}}}_{Kerr}|R\rangle \otimes |\alpha \rangle ={e}^{\frac{it}{\hslash }{H}_{Kerr}}|R\rangle \otimes |\alpha \rangle ={e}^{i\theta {N}_{R}{N}_{\alpha }}|R\rangle \otimes |\alpha \rangle =|R\rangle \otimes |\alpha {e}^{i\theta }\rangle ,\,\because {H}_{Kerr}=\hslash \chi {N}_{1}{N}_{2}$$

where *θ* = *χt* is the magnitude of conditional phase shift, and *t* is the interaction time in Kerr medium. Subsequently, when applied to the interaction, Eq. 2, of XKNL, the output state (signal-probe system) is changed to

3 $$\frac{1}{\sqrt{2}}({|R\rangle }^{1}+{|L\rangle }^{2})\otimes {|\alpha \rangle }^{a}\mathop{\to }\limits^{{\rm{X}}{\rm{K}}{\rm{N}}{\rm{L}}:{{\rm{U}}}_{Kerr}}\frac{1}{\sqrt{2}}({|R\rangle }^{1}\otimes {|\alpha {e}^{i\theta }\rangle }^{a}+{|L\rangle }^{2}\otimes {|\alpha \rangle }^{a}).\,[{\rm{O}}{\rm{U}}{\rm{T}}]$$

In Fig. 1, we can identify the photon state (signal system) according to the result of measuring ancillary system (probe beam) without measurement of signal system. This procedure is called quantum non-demolition measurement^12,25,41–46^.

### Interaction between a photon and QD within cavity (QD-cavity system)

**Figure 2:**
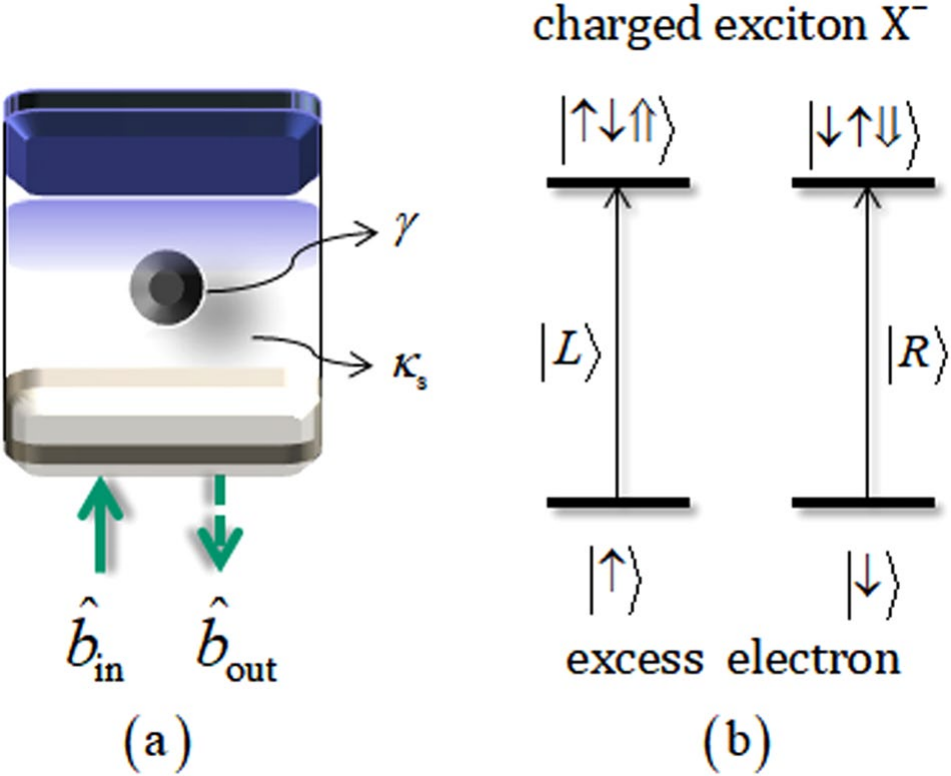
(**a**) Schematics of QD within a single-sided cavity (QD-cavity system): When this system interacts with photon (${\hat{{b}}}_{{\rm{in}}}$ and ${\hat{{b}}}_{{\rm{out}}}$), side leakage and energy decay occur from cavity mode and a negatively charged exciton at rates of *κ*_*s*_ and *γ*. (**b**) For optical transition (spin selection rule) in QD: $|L\rangle$ and $|R\rangle$ (photons) drive the transition of $|\uparrow \rangle \to |\uparrow \downarrow \Uparrow \rangle$ and $|\downarrow \rangle \to |\downarrow \uparrow \Downarrow \rangle$, respectively. $|\uparrow \rangle \equiv |+\,1/2\rangle ,\,|\downarrow \rangle \equiv |-1/2\rangle$ are spin states of the excess electron and $|\Uparrow \rangle ,\,|\Downarrow \rangle \,({J}_{z}=+\,3/2,-\,3/2)$ represent heavy-hole spin states.

QD-cavity system^4,6,7,23,47–54^ consists of a single charged QD confined in a single-sided cavity. Figure 2(a) schematically represents two GaAs/Al(Ga)As distributed Bragg reflectors [DBRs: the bottom DBR is partially reflective and the top one 100% reflective (single-sided cavity)] and transverse index guiding for the three-dimensional confinement of light. ${\hat{{b}}}_{{\rm{in}}}$ and ${\hat{{b}}}_{{\rm{out}}}$ are input and output field (photon) operators, *γ* is the decay rate of a negatively charged exciton (X^−^: consisting of two electrons bound to one hole^63^), and *κ*_*s*_ is the side leakage rate of optical cavity as described in Fig. 2(a). In Fig. 2(b), when the input photon of the left circular polarization $|L\rangle$ (right $|R\rangle$) is injected into the QD-cavity system, if the spin state of excess electron is in the state of $|\uparrow \rangle$ $(|\downarrow \rangle )$, the transition is created to the state of $|\uparrow \downarrow \Uparrow \rangle$ $(|\downarrow \uparrow \Downarrow \rangle )$ coupled the spin state with X^−^ (hot cavity) due to Pauli exclusion principle. Hot cavity of which the QD is coupled to the cavity can induce different reflectance, |*r*_h_(*ω*)|, and phase shift, ${\phi }_{{\rm{rh}}}(\omega )\equiv {\rm{\arg }}({R}_{{\rm{h}}}(\omega ))$, of the reflected photon, as follows:

4 $${R}_{{\rm{h}}}(\omega )\equiv |{r}_{{\rm{h}}}(\omega )|{e}^{i{\phi }_{{\rm{rh}}}(\omega )}=\frac{[i({\omega }_{{{\rm{X}}}^{-}}-\omega )+\gamma /2][i({\omega }_{c}-\omega )-\kappa /2+{\kappa }_{s}/2]+{g}^{2}}{[i({\omega }_{{{\rm{X}}}^{-}}-\omega )+\gamma /2][i({\omega }_{c}-\omega )+\kappa /2+{\kappa }_{s}/2]+{g}^{2}}=R(\omega ).$$

Otherwise, cold cavity of which the QD is uncoupled to cavity, i.e. $|L\rangle |\downarrow \rangle$ $(|R\rangle |\uparrow \rangle )$, the reflectance, |*r*_0_(*ω*)|, and phase shift, ${\phi }_{{\rm{r0}}}(\omega )\equiv {\rm{\arg }}({R}_{{\rm{0}}}(\omega ))$, of the reflected photon is given by

5 $${R}_{{\rm{0}}}(\omega )\equiv |{r}_{{\rm{0}}}(\omega )|{e}^{i{\phi }_{{\rm{r0}}}(\omega )}=\frac{i({\omega }_{c}-\omega )-\kappa /2+{\kappa }_{s}/2}{i({\omega }_{c}-\omega )+\kappa /2+{\kappa }_{s}/2},$$

where *R*_h_(*ω*) and *R*_0_(*ω*) are reflection coefficients, *ω*_*c*_ and *ω* are frequencies of cavity mode and external field, and *κ* and *g* are cavity decay rate and coupling strength (X^−^↔cavity mode). Here, we assume the steady state with ground state in QD, $\langle {\hat{\sigma }}_{Z}\rangle \approx -\,1$, and ${\omega }_{c}={\omega }_{{{\rm{X}}}^{-}}$ (${\omega }_{{{\rm{X}}}^{-}}$: the frequency of the dipole transition of X^−^) in weak approximation^64^ to the reflection operator $\hat{{\rm{R}}}(\omega )$ of the QD-cavity system^4,6,7,23,48–51^. And when having the side leakage rate as *κ*_*s*_ $\ll$ *κ* and coupling strength as *g* $\gg$ (*κ*, *γ*) with small *γ*(decay rate of X^−^)^47,65–68^ in the QD-cavity system, reflectances and phase shifts can be achieved to |*r*_0_(*ω*)| = |*r*_h_(*ω*)| ≈ 1, *ϕ*_rh_(*ω*) = 0, and *ϕ*_r0_(*ω*) = ±*π*/2 by adjusting frequencies (*ω* − *ω*_*c*_ = $\mp$*κ*/2). Therefore, we can express the reflection operator, $\hat{{\rm{R}}}$, for experimentally fixed parameters, as follow:

6 $$\hat{{\rm{R}}}\,\approx \,(|R\rangle \langle R|\otimes |\downarrow \rangle \langle \downarrow |+|L\rangle \langle L|\otimes |\uparrow \rangle \langle \uparrow |)-i(|R\rangle \langle R|\otimes |\uparrow \rangle \langle \uparrow |+|L\rangle \langle L|\otimes |\downarrow \rangle \langle \downarrow |),$$

where *g*/*κ* = 2.4, *κ*_*s*_ ≈ 0, *γ*/*κ* = 0.1, and *ω* − *ω*_*c*_ = *κ*/2. Subsequently, we will employ this interaction of the QD-cavity system (as parity gate) in our scheme to generate three-photon *W* state.

## Scheme of Generating Three-Photon *W* State Using XKNL and QD

**Figure 3:**
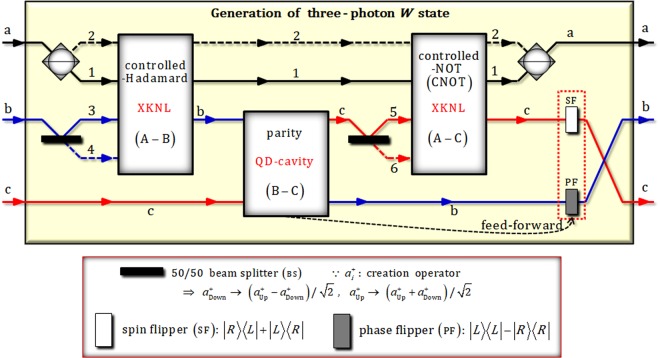
In our proposed scheme (generation of three-photon *W* state), critical components are nonlinearly optical (controlled and parity operations) gates using XKNLs and the QD-cavity system. Two controlled-unitary (controlled-Hadamard and CNOT) gates consist of weak XKNLs, qubus beams, and PNR measurements. Also, a QD within a single-sided optical cavity (the QD-cavity system) plays a role of parity operation which is implemented by the interaction between photons and a QD. Our scheme can generate three-photon entangled *W* state (final state) from three-photon product state (initial state) via nonlinearly optical gates and linear optical devices.

In Fig. 3, we propose an optical scheme for generating three-photon *W* state which has robust entanglement against loss of one photon using nonlinearly optical gates (XKNLs: controlled-unitary gates and QD-cavity system: parity gate) and linearly optical devices.

To describe the process of generating *W* state in our scheme, we assume the initial state (three-photon product state) as ${|H\rangle }_{{\rm{A}}}^{{\rm{a}}}\otimes {|R\rangle }_{{\rm{B}}}^{{\rm{b}}}\otimes {|H\rangle }_{{\rm{C}}}^{{\rm{c}}}$. As described in Fig. 3, after the initial state passes a CPBS and a BS, state of ${|{\phi }_{0}\rangle }_{{\rm{ABC}}}$ is given by

7 $${|H\rangle }_{{\rm{A}}}^{{\rm{a}}}\otimes {|R\rangle }_{{\rm{B}}}^{{\rm{b}}}\otimes {|H\rangle }_{{\rm{C}}}^{{\rm{c}}}\mathop{\to }\limits^{\mathrm{BS},\mathrm{CPBS}}{|{\phi }_{0}\rangle }_{{\rm{ABC}}}=\frac{1}{2}({|R\rangle }_{{\rm{A}}}^{1}{|R\rangle }_{{\rm{B}}}^{3}+{|R\rangle }_{{\rm{A}}}^{1}{|R\rangle }_{{\rm{B}}}^{4}+{|L\rangle }_{{\rm{A}}}^{2}{|R\rangle }_{{\rm{B}}}^{3}+{|L\rangle }_{{\rm{A}}}^{2}{|R\rangle }_{{\rm{B}}}^{4})\otimes {|H\rangle }_{{\rm{C}}}^{{\rm{c}}}.$$

### Controlled-Hadamard gate (XKNLs)

**Figure 4:**
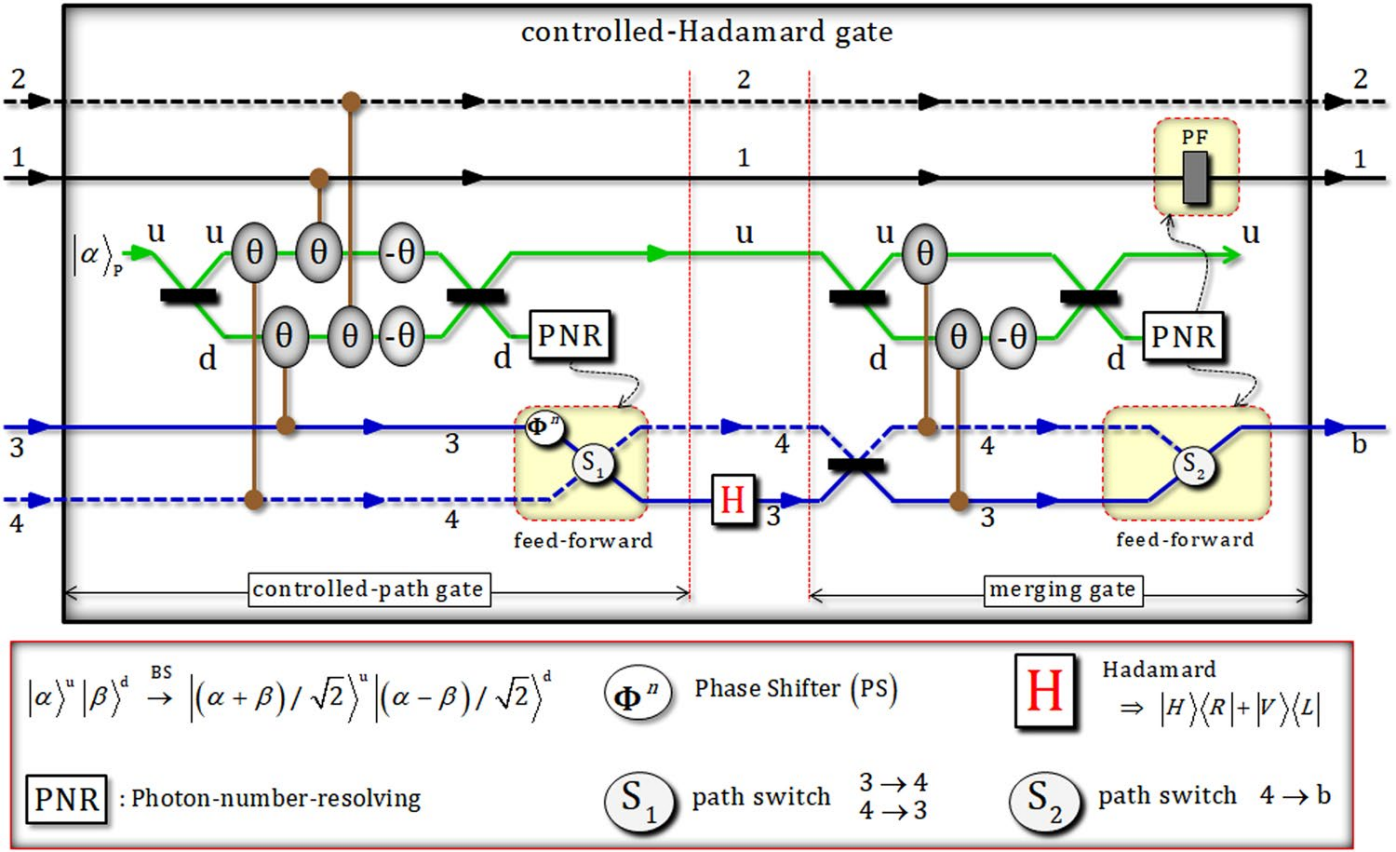
The controlled-Hadamard gate: This gate consists of controlled-path and merging-path gates using weak XKNLs, qubus beams, and PNR measurements. Paths of photons A and B are particularly arranged after a controlled-path gate. Hadamard operation is then performed on path 3 of photon B. Subsequently, the merging-path gate can merge to single path b of photon B. Finally, entire gates (controlled-path and merging-path) and an operation (Hadamard) can facilitate the controlled-Hadamard operation in terms of the state of two photons A and B. Also, probe beams (coherent state: *α*) used in the controlled-path gate can be recycled to utilize probe beams of merging gate.

Two photons (A and B) in the state, ${|{\phi }_{0}\rangle }_{{\rm{ABC}}}$, are injected to controlled-Hadamard gate consisting of controlled-path and merging-path gates by interaction of XKNLs (Sec. 2) using XKNLs, qubus beams, and PNR measurements, as shown in Fig. 4. After the state ${|{\phi }_{0}\rangle }_{{\rm{ABC}}}$ passes through the controlled-path gate in Fig. 4, the state, ${|{\phi }_{1}\rangle }_{{\rm{ABC}}\otimes {\rm{P}}}$ (pre-measurement) can be given by

8 $$\begin{array}{c}{|{\varphi }_{0}\rangle }_{{\rm{ABC}}}\mathop{\to }\limits^{\mathrm{controlled}-\mathrm{path}\,{\rm{gate}}}\,{|{\varphi }_{1}\rangle }_{{\rm{ABC}}\otimes {\rm{P}}}=\frac{1}{\sqrt{2}}[\frac{1}{\sqrt{2}}({|R\rangle }_{{\rm{A}}}^{1}{|R\rangle }_{{\rm{B}}}^{3}+{|L\rangle }_{{\rm{A}}}^{2}{|R\rangle }_{{\rm{B}}}^{4})\otimes {|H\rangle }_{{\rm{C}}}^{{\rm{c}}}\otimes {|\alpha \rangle }_{{\rm{P}}}^{{\rm{u}}}{|0\rangle }_{{\rm{P}}}^{{\rm{d}}}\\ \,+\,{e}^{-\frac{{(\alpha \sin {\rm{\theta }})}^{2}}{2}}\sum _{n=0}^{\infty }\frac{{(i\alpha \sin {\rm{\theta }})}^{n}}{\sqrt{n!}}\{\frac{1}{\sqrt{2}}({|R\rangle }_{{\rm{A}}}^{1}{|R\rangle }_{{\rm{B}}}^{4}\\ \,+{(-1)}^{n}{|L\rangle }_{{\rm{A}}}^{2}{|R\rangle }_{{\rm{B}}}^{3})\otimes {|H\rangle }_{{\rm{C}}}^{{\rm{c}}}\}\otimes {|\alpha \,\cos \,{\rm{\theta }}\rangle }_{{\rm{P}}}^{{\rm{u}}}{|n\rangle }_{{\rm{P}}}^{{\rm{d}}}],\end{array}$$

where $|\pm i\alpha \,\sin \,\theta \rangle ={e}^{-\frac{{(\alpha \sin \theta )}^{2}}{2}}\sum _{n=0}^{\infty }\frac{{(\pm i\alpha \sin \theta )}^{n}}{\sqrt{n!}}|n\rangle$ for *α* ∈ **R**. When performing PNR measurement on path d of probe beam (coherent state), if the result (photon number) is ${|0\rangle }_{{\rm{P}}}^{{\rm{d}}}$ (photon number: zero or dark detection), the output state, ${|{\phi }_{1}\rangle }_{{\rm{ABC}}}$, of controlled-path gate will be as ${|{\phi }_{1}\rangle }_{{\rm{ABC}}}=\frac{1}{\sqrt{2}}({|R\rangle }_{{\rm{A}}}^{1}{|R\rangle }_{{\rm{B}}}^{3}+{|L\rangle }_{{\rm{A}}}^{2}{|R\rangle }_{{\rm{B}}}^{4})\otimes {|H\rangle }_{{\rm{C}}}^{{\rm{c}}}$. Otherwise, if the result is the state ${|n\rangle }_{{\rm{P}}}^{{\rm{d}}}$ (*n* ≠ 0), the output state can be transformed to state ${|{\phi }_{1}\rangle }_{{\rm{ABC}}}$ (the case of zero photon) by feed-forward (PS and path switch, S_1_) according to the result (photon number *n*) on path d. Then, as described in Fig. 4, Hadamard operator performs path 3 of photon B in the state of ${|{\phi }_{1}\rangle }_{{\rm{ABC}}}$, as follows:

9 $$\begin{array}{ccc}{|{\phi }_{1}\rangle }_{{\rm{A}}{\rm{B}}{\rm{C}}}\mathop{\to }\limits^{{\rm{H}}}{|{\phi }_{2}\rangle }_{{\rm{A}}{\rm{B}}{\rm{C}}} & = & \frac{1}{\sqrt{2}}({|R\rangle }_{{\rm{A}}}^{1}{|H\rangle }_{{\rm{B}}}^{3}+{|L\rangle }_{{\rm{A}}}^{2}{|R\rangle }_{{\rm{B}}}^{4})\otimes {|H\rangle }_{{\rm{C}}}^{{\rm{c}}}\\  & = & \frac{1}{2}({|R\rangle }_{{\rm{A}}}^{1}{|R\rangle }_{{\rm{B}}}^{3}+{|R\rangle }_{{\rm{A}}}^{1}{|L\rangle }_{{\rm{B}}}^{3}+\sqrt{2}{|L\rangle }_{{\rm{A}}}^{2}{|R\rangle }_{{\rm{B}}}^{4})\otimes {|H\rangle }_{{\rm{C}}}^{{\rm{c}}}.\end{array}$$

Before the merging-path gate, we can see the method to recycle probe beam (coherent state), which was utilized. In Eq. 8, after PNR measurement, the probe beam (coherent state) still remains to ${|\alpha \rangle }_{{\rm{P}}}^{{\rm{u}}}$ or ${|\alpha \,\cos \,{\rm{\theta }}\rangle }_{{\rm{P}}}^{{\rm{u}}}$ due to PNR measurement in controlled-path gate because PNR measurement is only applied to path d. Thus, we can recycle the remaining (used) probe beam on path u for the probe beam of merging-path gate. Let us assume to choose the remaining probe beam, ${|\alpha \,\cos \,{\rm{\theta }}\rangle }_{{\rm{P}}}^{{\rm{u}}}\equiv {|\beta \rangle }_{{\rm{P}}}^{{\rm{u}}}$, after the measurement of controlled-path gate. Thus, the state ${|{\phi }_{3}\rangle }_{{\rm{ABC}}\otimes {\rm{P}}}$ (pre-measurement) is transformed by merging-path gate in Fig. 4, as follows:

10 $$\begin{array}{c}{|{\varphi }_{2}\rangle }_{{\rm{ABC}}}\mathop{\to }\limits^{\mathrm{merging}-\mathrm{path}\,{\rm{gate}}}{|{\varphi }_{3}\rangle }_{{\rm{ABC}}\otimes {\rm{P}}}\\ \,=\frac{1}{\sqrt{2}}[\frac{1}{2}(\,-\,{|R\rangle }_{{\rm{A}}}^{1}{|R\rangle }_{{\rm{B}}}^{3}-{|R\rangle }_{{\rm{A}}}^{1}{|L\rangle }_{{\rm{B}}}^{3}+\sqrt{2}{|L\rangle }_{{\rm{A}}}^{2}{|R\rangle }_{{\rm{B}}}^{3})\otimes {|H\rangle }_{{\rm{C}}}^{{\rm{c}}}\otimes {|\beta \rangle }_{{\rm{P}}}^{{\rm{u}}}{|0\rangle }_{{\rm{P}}}^{{\rm{d}}}\\ \,+\,{e}^{-\frac{{(\alpha \sin {\rm{\theta }})}^{2}}{2}}\sum _{n=0}^{\infty }\frac{{(i\alpha \sin {\rm{\theta }})}^{n}}{\sqrt{n!}}\{\frac{1}{2}({|R\rangle }_{{\rm{A}}}^{1}{|R\rangle }_{{\rm{B}}}^{4}+{|R\rangle }_{{\rm{A}}}^{1}{|L\rangle }_{{\rm{B}}}^{4}\\ \,+\sqrt{2}{|L\rangle }_{{\rm{A}}}^{2}{|R\rangle }_{{\rm{B}}}^{4})\otimes {|H\rangle }_{{\rm{C}}}^{{\rm{c}}}\}\otimes {|\beta \,\cos \,{\rm{\theta }}\rangle }_{{\rm{P}}}^{{\rm{u}}}{|n\rangle }_{{\rm{P}}}^{{\rm{d}}}],\end{array}$$

where ${|\beta \,\cos \,{\rm{\theta }}\rangle }_{{\rm{P}}}^{{\rm{u}}}={|(\alpha \,\cos \,{\rm{\theta }})\cos \,{\rm{\theta }}\rangle }_{{\rm{P}}}^{{\rm{u}}}$. If the result of PNR measurement on path b is in the ${|0\rangle }_{{\rm{P}}}^{{\rm{d}}}$ (zero photon), the output state, ${|{\phi }_{3}\rangle }_{{\rm{ABC}}}$, can be given by ${|{\phi }_{3}\rangle }_{{\rm{ABC}}}=\frac{1}{2}(\,-\,{|R\rangle }_{{\rm{A}}}^{1}{|R\rangle }_{{\rm{B}}}^{{\rm{b}}}-{|R\rangle }_{{\rm{A}}}^{1}{|L\rangle }_{{\rm{B}}}^{{\rm{b}}}+\sqrt{2}{|L\rangle }_{{\rm{A}}}^{2}{|R\rangle }_{{\rm{B}}}^{{\rm{b}}})\otimes {|H\rangle }_{{\rm{C}}}^{{\rm{c}}}$. Also, if the result is in the state ${|n\rangle }_{{\rm{P}}}^{{\rm{d}}}$ (*n* ≠ 0), the output state can be transformed to state ${|{\phi }_{3}\rangle }_{{\rm{ABC}}}$ (the case of zero photon) by feed-forward (PF and path switch, S_2_) in Fig. 4 due to the result (photon number *n*) on path d. Error probabilities ${{\rm{P}}}_{{\rm{err}}}^{{\rm{CP}}}$ and ${{\rm{P}}}_{{\rm{err}}}^{{\rm{MP}}}$ of controlled-path and merging-path gates in Fig. 4 can be calculated by probabilities to measure ${|0\rangle }_{{\rm{P}}}^{{\rm{d}}}$ (zero photon) in ${|\pm i\alpha \,\sin \,\theta \rangle }_{{\rm{P}}}^{{\rm{d}}}$ (controlled-path gate) and ${|\beta \,\sin \,\theta \rangle }_{{\rm{P}}}^{{\rm{d}}}$ merging-path gate) on path d of qubus beams (Fig. 4), as follows:

11 $$\begin{array}{ccc}{{\rm{P}}}_{{\rm{e}}{\rm{r}}{\rm{r}}}^{{\rm{C}}{\rm{P}}}=\frac{1}{2}\exp (\,-\,{\alpha }^{2}{\sin }^{2}\theta ) & \approx  & \frac{1}{2}\exp (\,-\,{\alpha }^{2}{\theta }^{2}),\\ {{\rm{P}}}_{{\rm{e}}{\rm{r}}{\rm{r}}}^{{\rm{M}}{\rm{P}}}=\frac{1}{2}\exp (\,-\,{\beta }^{2}{\sin }^{2}\theta ) & = & \frac{1}{2}\exp (\,-\,({\alpha }^{2}{\cos }^{2}\theta ){\sin }^{2}\theta )\,\,\because \beta \equiv \alpha \,\cos \,\theta \\  & \approx  & \frac{1}{2}\exp (\,-\,{\alpha }^{2}{\theta }^{2}),\end{array}$$

where ${\alpha }^{2}{\sin }^{2}\theta \approx {\alpha }^{2}{\theta }^{2}$ and ${\alpha }^{2}{\cos }^{2}\theta \approx {\alpha }^{2}$ for *α* ≫ 10 and *θ* ≪ 0.1. If parameters (*α*: amplitude of coherent state and *θ*: magnitude of conditional phase shift) are fixed as *αθ* = 2.5 ($\because \theta  < {10}^{-2}$), error probabilities (${{\rm{P}}}_{{\rm{err}}}^{{\rm{CP}}}$ and ${{\rm{P}}}_{{\rm{err}}}^{{\rm{MP}}}$) can be obtained as ${{\rm{P}}}_{{\rm{err}}}^{{\rm{CP}}}\approx {{\rm{P}}}_{{\rm{err}}}^{{\rm{MP}}} < {10}^{-3}$. Moreover, when we increase the amplitude of coherent state with fixed *θ* < 10^−2^ in controlled-path and merging-path gates, error probabilities (${{\rm{P}}}_{{\rm{err}}}^{{\rm{CP}}}$ and ${{\rm{P}}}_{{\rm{err}}}^{{\rm{MP}}}$) can be approaching zero. Consequently, we can see the operation of controlled-Hadamard (controlled-path and merging-path gates) by compare input state, ${|{\phi }_{0}\rangle }_{{\rm{ABC}}}$, and output state, ${|{\phi }_{3}\rangle }_{{\rm{ABC}}}$, as follows:

12 $$\begin{array}{ccc}{|{\phi }_{0}\rangle }_{{\rm{A}}{\rm{B}}{\rm{C}}} & = & \frac{1}{2}({|R\rangle }_{{\rm{A}}}^{1}{|R\rangle }_{{\rm{B}}}^{3}+{|R\rangle }_{{\rm{A}}}^{1}{|R\rangle }_{{\rm{B}}}^{4}+{|L\rangle }_{{\rm{A}}}^{2}{|R\rangle }_{{\rm{B}}}^{3}+{|L\rangle }_{{\rm{A}}}^{2}{|R\rangle }_{{\rm{B}}}^{4})\otimes {|H\rangle }_{{\rm{C}}}^{{\rm{c}}}\\ \mathop{\to }\limits^{{\rm{c}}{\rm{o}}{\rm{n}}{\rm{t}}{\rm{r}}{\rm{o}}{\rm{l}}{\rm{l}}{\rm{e}}{\rm{d}}-{\rm{H}}{\rm{a}}{\rm{d}}{\rm{a}}{\rm{m}}{\rm{a}}{\rm{r}}{\rm{d}}} & \\ \to {|{\phi }_{3}\rangle }_{{\rm{A}}{\rm{B}}{\rm{C}}} & = & \frac{1}{2}(\,-\,{|R\rangle }_{{\rm{A}}}^{1}{|R\rangle }_{{\rm{B}}}^{{\rm{b}}}-{|R\rangle }_{{\rm{A}}}^{1}{|L\rangle }_{{\rm{B}}}^{{\rm{b}}}+\sqrt{2}{|L\rangle }_{{\rm{A}}}^{2}{|R\rangle }_{{\rm{B}}}^{{\rm{b}}})\otimes {|H\rangle }_{{\rm{C}}}^{{\rm{c}}}\\  & = & \frac{1}{2}(\,-\,{|R\rangle }_{{\rm{A}}}^{1}{|H\rangle }_{{\rm{B}}}^{{\rm{b}}}-{|R\rangle }_{{\rm{A}}}^{1}{|H\rangle }_{{\rm{B}}}^{{\rm{b}}}+{|L\rangle }_{{\rm{A}}}^{2}{|R\rangle }_{{\rm{B}}}^{{\rm{b}}}+{|L\rangle }_{{\rm{A}}}^{2}{|R\rangle }_{{\rm{B}}}^{{\rm{b}}})\otimes {|H\rangle }_{{\rm{C}}}^{{\rm{c}}}.\end{array}$$

From this equation, we can confirm that the input state, ${|{\phi }_{0}\rangle }_{{\rm{ABC}}}$, is transformed to the output state, ${|{\phi }_{3}\rangle }_{{\rm{ABC}}}$ by controlled-Hadamard gate in Fig. 4. If a photon A is in the state ${|R\rangle }_{{\rm{A}}}$, the operation of Hadamard is applied to photon B.

### Parity gate (the QD-cavity system)

**Figure 5:**
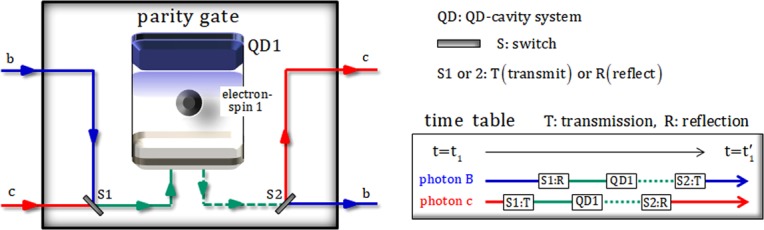
The parity gate: This parity gate utilizes the interaction which can be described by reflection operator $\hat{{\rm{R}}}$ in Eq. 6, between two photons (B and C) and an electron 1 in QD1 (QD-cavity system) for *g*/*κ* = 2.4, *κ*_*s*_ = 0, *γ*/*κ* = 0.1, and *ω* − *ω*_*c*_ = *κ*/2. This gate can arrange polarizations of two photons B and C, such as $\{|R\rangle |L\rangle ,\,|L\rangle |R\rangle \}$ (odd) and $\{|R\rangle |R\rangle ,\,|L\rangle |L\rangle \}$ (even). Switches (S1 and S2) are also controlled to transmit or reflect photons (B and C), due to a time table.

As shown in Fig. 5, the QD-cavity system (QD1) confined in a single-sided cavity sequentially can interact with two photons (B and C) in the state of ${|{\phi }_{3}\rangle }_{{\rm{ABC}}}$. For interaction of photons-electron in the parity gate using the QD-cavity system, we prepare an excess electron-spin state as ${|{+}_{e}\rangle }_{1}$ [$|{\pm }_{e}\rangle \equiv (|\uparrow \rangle \pm |\downarrow \rangle )/\sqrt{2}$]. Subsequently, photons and electron 1 of the input state, ${|{+}_{e}\rangle }_{1}\otimes {|{\phi }_{3}\rangle }_{{\rm{ABC}}}$, will sequentially interact in the QD-cavity system, according to the time table shown in Fig. 5. After interactions in the QD-cavity system, the output state (photons-electron) is given by

13 $$\begin{array}{c}{|{+}_{e}\rangle }_{1}\otimes {|{\phi }_{3}\rangle }_{{\rm{A}}{\rm{B}}{\rm{C}}}\mathop{\to }\limits^{{\rm{p}}{\rm{a}}{\rm{r}}{\rm{i}}{\rm{t}}{\rm{y}}\,{\rm{g}}{\rm{a}}{\rm{t}}{\rm{e}}}{|{\phi }_{4}\rangle }_{1{\rm{A}}{\rm{C}}{\rm{B}}}\\ =\frac{-i}{\sqrt{2}}{|{+}_{e}\rangle }_{1}[\frac{1}{2}(\,-\,{|R\rangle }_{{\rm{A}}}^{1}{|L\rangle }_{{\rm{C}}}^{{\rm{c}}}{|R\rangle }_{{\rm{B}}}^{{\rm{b}}}-{|R\rangle }_{{\rm{A}}}^{1}{|R\rangle }_{{\rm{C}}}^{{\rm{c}}}{|L\rangle }_{{\rm{B}}}^{{\rm{b}}}+\sqrt{2}{|L\rangle }_{{\rm{A}}}^{2}{|L\rangle }_{{\rm{C}}}^{{\rm{c}}}{|R\rangle }_{{\rm{B}}}^{{\rm{b}}})]\\ +\,\frac{1}{\sqrt{2}}{|{-}_{e}\rangle }_{1}[\frac{1}{2}({|R\rangle }_{{\rm{A}}}^{1}{|R\rangle }_{{\rm{C}}}^{{\rm{c}}}{|R\rangle }_{{\rm{B}}}^{{\rm{b}}}-{|R\rangle }_{{\rm{A}}}^{1}{|L\rangle }_{{\rm{C}}}^{{\rm{c}}}{|L\rangle }_{{\rm{B}}}^{{\rm{b}}}-\sqrt{2}{|L\rangle }_{{\rm{A}}}^{2}{|R\rangle }_{{\rm{C}}}^{{\rm{c}}}{|R\rangle }_{{\rm{B}}}^{{\rm{b}}})],\end{array}$$

where interactions between photons and an electron 1 can be expressed by the reflection operator $\hat{{\rm{R}}}$ in Eq. 6 for *g*/*κ* = 2.4, *κ*_*s*_ = 0, *γ*/*κ* = 0.1, and *ω* − *ω*_*c*_ = *κ*/2. Based on the result, ${|{+}_{e}\rangle }_{1}$ or ${|{-}_{e}\rangle }_{1}$, of measurement in electron-spin state 1, we can know the output state ${|{\phi }_{4}^{+}\rangle }_{{\rm{ACB}}}$ (odd: ${|RL\rangle }_{{\rm{CB}}}$, ${|LR\rangle }_{{\rm{CB}}}$) or ${|{\phi }_{4}^{-}\rangle }_{{\rm{ACB}}}$ (even: ${|RR\rangle }_{{\rm{CB}}}$, ${|LL\rangle }_{{\rm{CB}}}$), of photons A, B, and C. Here, let us suppose that the output state from parity gate is in the state ${|{\phi }_{4}^{+}\rangle }_{{\rm{ACB}}}$ according to the result ${|{+}_{e}\rangle }_{1}$ of an electron-spin state 1. As described in Fig. 3, after the state, ${|{\phi }_{4}^{+}\rangle }_{{\rm{ACB}}}$, passes a BS, the state ${|{\phi }_{5}^{+}\rangle }_{{\rm{ACB}}}$ is given by

14 $$\begin{array}{c}{|{\phi }_{4}^{+}\rangle }_{{\rm{A}}{\rm{C}}{\rm{B}}}\mathop{\to }\limits^{{\rm{B}}{\rm{S}}}{|{\phi }_{5}^{+}\rangle }_{{\rm{A}}{\rm{C}}{\rm{B}}}\\ =\frac{1}{\sqrt{2}}[\frac{1}{2}(\,-\,{|R\rangle }_{{\rm{A}}}^{1}{|L\rangle }_{{\rm{C}}}^{5}{|R\rangle }_{{\rm{B}}}^{{\rm{b}}}-{|R\rangle }_{{\rm{A}}}^{1}{|R\rangle }_{{\rm{C}}}^{5}{|L\rangle }_{{\rm{B}}}^{{\rm{b}}}+\sqrt{2}{|L\rangle }_{{\rm{A}}}^{2}{|L\rangle }_{{\rm{C}}}^{5}{|R\rangle }_{{\rm{B}}}^{{\rm{b}}})\\ +\frac{1}{2}(\,-\,{|R\rangle }_{{\rm{A}}}^{1}{|L\rangle }_{{\rm{C}}}^{6}{|R\rangle }_{{\rm{B}}}^{{\rm{b}}}-{|R\rangle }_{{\rm{A}}}^{1}{|R\rangle }_{{\rm{C}}}^{6}{|L\rangle }_{{\rm{B}}}^{{\rm{b}}}+\sqrt{2}{|L\rangle }_{{\rm{A}}}^{2}{|L\rangle }_{{\rm{C}}}^{6}{|R\rangle }_{{\rm{B}}}^{{\rm{b}}})].\end{array}$$

### CNOT gate (XKNLs)

**Figure 6:**
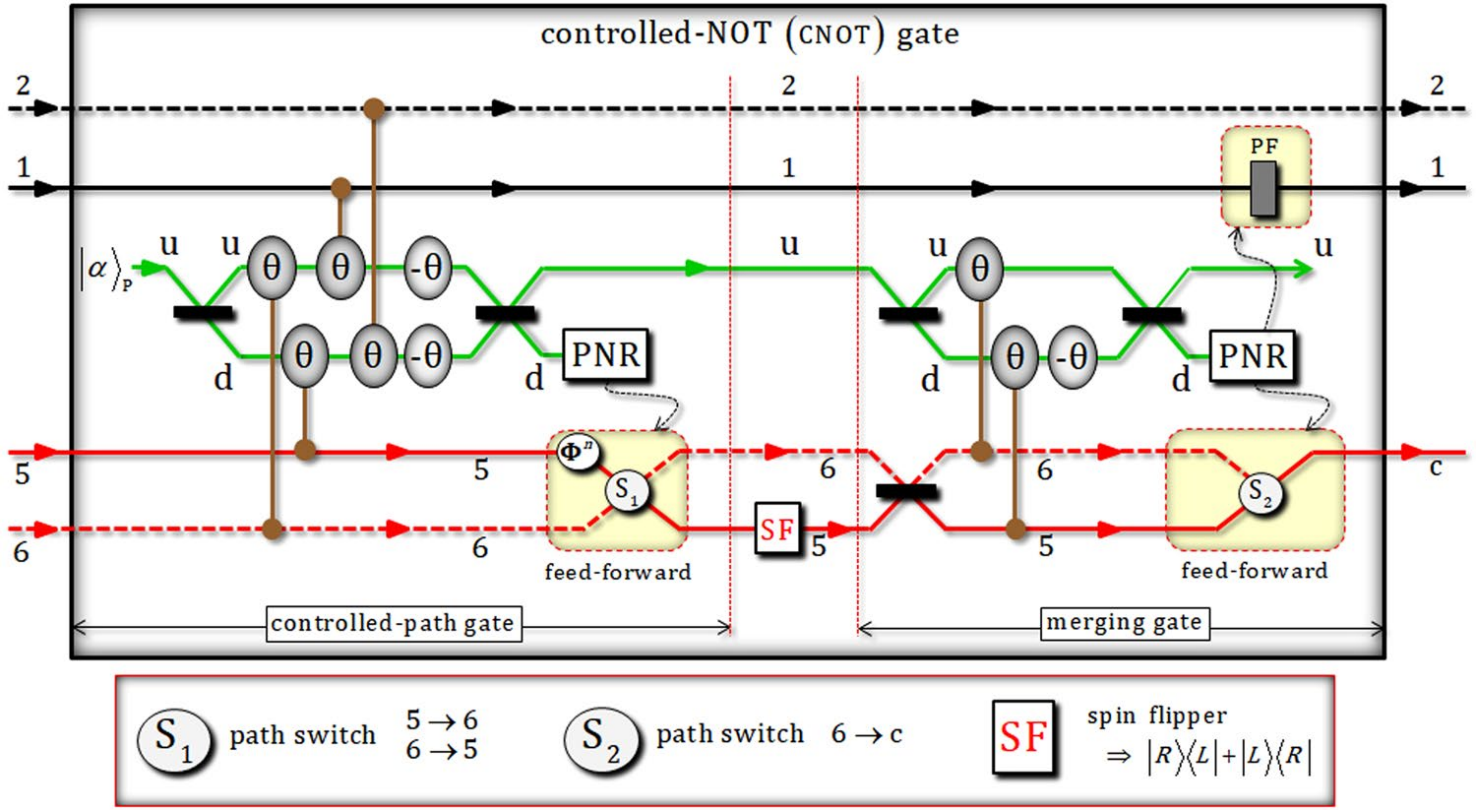
CNOT gate: This gate is constructively the same as controlled-Hadamard gate in Fig. 4. The SF operator between controlled-path and merging-path gates plays the role of NOT gate. Similar to controlled-Hadamard gate, probe beams (coherent state: *α*) used in controlled-path gate can also be recycled to be utilized as probe beams of merging gate.

In Fig. 6, two photons (A and C) in the state, ${|{\phi }_{5}^{+}\rangle }_{{\rm{ACB}}}$, are injected to CNOT gate via XKNLs, qubus beams, and PNR measurements. As described in Fig. 6, construction of this gate is almost identical to that of controlled-Hadamard gate. CNOT gate in Fig. 6 is also comprised of controlled-path and merging-path gates. It performs PNR measurement on path d in probe beam and feed-forwards for the transformation of output state. Besides, it can recycle the probe beam (unmeasured) on path u. The state of ${|{\phi }_{6}^{+}\rangle }_{{\rm{ACB}}\otimes {\rm{P}}}$ can be transformed from the state of ${|{\phi }_{5}^{+}\rangle }_{{\rm{ACB}}}$ by controlled-path gate in Fig. 6, before PNR measurement, as follows:

15 $$\begin{array}{l}{|{\phi }_{5}^{+}\rangle }_{{\rm{ACB}}}\mathop{\to }\limits^{\mathrm{controlled}-\mathrm{path}\,{\rm{gate}}}\\ \begin{array}{rcl}\to {|{\phi }_{6}^{+}\rangle }_{{\rm{ACB}}\otimes {\rm{P}}} & = & \frac{1}{\sqrt{2}}[\frac{1}{2}(-{|R\rangle }_{{\rm{A}}}^{1}{|L\rangle }_{{\rm{C}}}^{{\rm{5}}}{|R\rangle }_{{\rm{B}}}^{{\rm{b}}}-{|R\rangle }_{{\rm{A}}}^{1}{|R\rangle }_{{\rm{C}}}^{{\rm{5}}}{|L\rangle }_{{\rm{B}}}^{{\rm{b}}}+\sqrt{2}{|L\rangle }_{{\rm{A}}}^{2}{|L\rangle }_{{\rm{C}}}^{{\rm{6}}}{|R\rangle }_{{\rm{B}}}^{{\rm{b}}})\\  &  & \otimes \,{|\alpha \rangle }_{{\rm{P}}}^{{\rm{u}}}{|0\rangle }_{{\rm{P}}}^{{\rm{d}}}\\  &  & +\,{e}^{-\frac{{(\alpha \sin {\rm{\theta }})}^{2}}{2}}\sum _{n=0}^{\infty }\frac{{(i\alpha \sin {\rm{\theta }})}^{n}}{\sqrt{n!}}\{\frac{1}{2}(-{|R\rangle }_{{\rm{A}}}^{1}{|L\rangle }_{{\rm{C}}}^{{\rm{6}}}{|R\rangle }_{{\rm{B}}}^{{\rm{b}}}-{|R\rangle }_{{\rm{A}}}^{1}{|R\rangle }_{{\rm{C}}}^{{\rm{6}}}{|L\rangle }_{{\rm{B}}}^{{\rm{b}}}\\  &  & +\,{(-1)}^{n}\sqrt{2}{|L\rangle }_{{\rm{A}}}^{2}{|L\rangle }_{{\rm{C}}}^{{\rm{5}}}{|R\rangle }_{{\rm{B}}}^{{\rm{b}}})\}\otimes {|\alpha \,\cos \,{\rm{\theta }}\rangle }_{{\rm{P}}}^{{\rm{u}}}{|n\rangle }_{{\rm{P}}}^{{\rm{d}}}].\end{array}\end{array}$$

After PNR measurement, as described in Fig. 6, the output state ${|{\phi }_{6}^{+}\rangle }_{{\rm{ACB}}}$ of controlled-path gate can be obtained as ${|{\phi }_{6}^{+}\rangle }_{{\rm{ACB}}}=(-{|R\rangle }_{{\rm{A}}}^{1}{|L\rangle }_{{\rm{C}}}^{{\rm{5}}}{|R\rangle }_{{\rm{B}}}^{{\rm{b}}}-{|R\rangle }_{{\rm{A}}}^{1}{|R\rangle }_{{\rm{C}}}^{{\rm{5}}}{|L\rangle }_{{\rm{B}}}^{{\rm{b}}}+\sqrt{2}{|L\rangle }_{{\rm{A}}}^{2}{|L\rangle }_{{\rm{C}}}^{{\rm{6}}}{|R\rangle }_{{\rm{B}}}^{{\rm{b}}})/2$ by feed-forward or not, due to result of PNR measurement. Then, SF (spin flipper) operator performs path 5 of photon C in the state of ${|{\phi }_{6}^{+}\rangle }_{{\rm{ACB}}}$, as follows:

16 $${|{\phi }_{6}^{+}\rangle }_{{\rm{ACB}}}\mathop{\to }\limits^{{\rm{SF}}}{|{\phi }_{7}^{+}\rangle }_{{\rm{ACB}}}=\frac{1}{2}(-{|R\rangle }_{{\rm{A}}}^{1}{|R\rangle }_{{\rm{C}}}^{{\rm{5}}}{|R\rangle }_{{\rm{B}}}^{{\rm{b}}}-{|R\rangle }_{{\rm{A}}}^{1}{|L\rangle }_{{\rm{C}}}^{{\rm{5}}}{|L\rangle }_{{\rm{B}}}^{{\rm{b}}}+\sqrt{2}{|L\rangle }_{{\rm{A}}}^{2}{|L\rangle }_{{\rm{C}}}^{{\rm{6}}}{|R\rangle }_{{\rm{B}}}^{{\rm{b}}}).$$

Subsequently, we can recycle the remaining (used) probe beam on path u as in the controlled-Hadamard gate. Thus, we also assume the remaining probe beam as ${|\alpha \,\cos \,{\rm{\theta }}\rangle }_{{\rm{P}}}^{{\rm{u}}}\equiv {|\beta \rangle }_{{\rm{P}}}^{{\rm{u}}}$. After the state ${|{\phi }_{7}^{+}\rangle }_{{\rm{ACB}}}$ passes through the controlled-merging gate in Fig. 6, the state ${|{\phi }_{8}^{+}\rangle }_{{\rm{ACB}}\otimes {\rm{P}}}$ (pre-measurement), will be given by

17 $$\begin{array}{c}{|{\phi }_{7}^{+}\rangle }_{{\rm{A}}{\rm{C}}{\rm{B}}}\mathop{\to }\limits^{{\rm{m}}{\rm{e}}{\rm{r}}{\rm{g}}{\rm{i}}{\rm{n}}{\rm{g}}-{\rm{p}}{\rm{a}}{\rm{t}}{\rm{h}}\,{\rm{g}}{\rm{a}}{\rm{t}}{\rm{e}}}\\ \to {|{\phi }_{8}^{+}\rangle }_{{\rm{A}}{\rm{B}}{\rm{C}}\otimes {\rm{P}}}=\frac{1}{\sqrt{2}}[\frac{1}{2}({|R\rangle }_{{\rm{A}}}^{1}{|R\rangle }_{{\rm{C}}}^{5}{|R\rangle }_{{\rm{B}}}^{{\rm{b}}}+{|R\rangle }_{{\rm{A}}}^{1}{|L\rangle }_{{\rm{C}}}^{5}{|L\rangle }_{{\rm{B}}}^{{\rm{b}}}+\sqrt{2}{|L\rangle }_{{\rm{A}}}^{2}{|L\rangle }_{{\rm{C}}}^{5}{|R\rangle }_{{\rm{B}}}^{{\rm{b}}})\otimes {|\beta \rangle }_{{\rm{P}}}^{{\rm{u}}}{|0\rangle }_{{\rm{P}}}^{{\rm{d}}}\\ +{e}^{-\frac{{(\alpha \sin \theta )}^{2}}{2}}\sum _{n=0}^{{\rm{\infty }}}\frac{{(i\alpha \sin \theta )}^{n}}{\sqrt{n!}}\{\frac{1}{2}(\,-\,{|R\rangle }_{{\rm{A}}}^{1}{|R\rangle }_{{\rm{C}}}^{6}{|R\rangle }_{{\rm{B}}}^{{\rm{b}}}-{|R\rangle }_{{\rm{A}}}^{1}{|L\rangle }_{{\rm{C}}}^{6}{|L\rangle }_{{\rm{B}}}^{{\rm{b}}}+\sqrt{2}{|L\rangle }_{{\rm{A}}}^{2}{|L\rangle }_{{\rm{C}}}^{6}{|R\rangle }_{{\rm{B}}}^{{\rm{b}}})\}\otimes {|\beta \,\cos \,\theta \rangle }_{{\rm{P}}}^{{\rm{u}}}{|n\rangle }_{{\rm{P}}}^{{\rm{d}}}],\end{array}$$

where ${|\beta \,\cos \,{\rm{\theta }}\rangle }_{{\rm{P}}}^{{\rm{u}}}={|(\alpha \,\cos \,{\rm{\theta }})\cos \,{\rm{\theta }}\rangle }_{{\rm{P}}}^{{\rm{u}}}$. After PNR measurement, as described in Fig. 6, the output state ${|{\phi }_{8}^{+}\rangle }_{{\rm{ACB}}}$ of merging-path gate can be obtained as ${|{\phi }_{8}^{+}\rangle }_{{\rm{ACB}}}=({|R\rangle }_{{\rm{A}}}^{1}{|R\rangle }_{{\rm{C}}}^{{\rm{c}}}{|R\rangle }_{{\rm{B}}}^{{\rm{b}}}+{|R\rangle }_{{\rm{A}}}^{1}{|L\rangle }_{{\rm{C}}}^{{\rm{c}}}{|L\rangle }_{{\rm{B}}}^{{\rm{b}}}+\sqrt{2}{|L\rangle }_{{\rm{A}}}^{2}{|L\rangle }_{{\rm{C}}}^{{\rm{c}}}{|R\rangle }_{{\rm{B}}}^{{\rm{b}}})/2$ by feed-forward or not, due to result of PNR measurement. Error probabilities ${{\rm{P}}}_{{\rm{err}}}^{{\rm{CP}}}$ and ${{\rm{P}}}_{{\rm{err}}}^{{\rm{MP}}}$ of controlled-path and merging-path gates in Fig. 6, are the same as those with the aforementioned gates, Eq. 11, of controlled-Hadamard gate in Fig. 4. Finally, by comparing with input state, ${|{\phi }_{5}^{+}\rangle }_{{\rm{ACB}}}$ in Eq. 14 and output state, ${|{\phi }_{8}^{+}\rangle }_{{\rm{ACB}}}$, we can confirm the operation of CNOT gate. If a photon A is in the state ${|R\rangle }_{{\rm{A}}}$, the operation of spin flip is applied to photon C.

Subsequently, as described in Fig. 3, a photon A in the state ${|{\phi }_{8}^{+}\rangle }_{{\rm{ACB}}}$ passes through a CPBS. Then SF is operated to a photon C by feed-forward (red-dotted box in Fig. 3) according to the result of parity gate in Fig. 5 (we assumed the result of electron-spin state as ${|{+}_{e}\rangle }_{1}$). Finally, after crossing paths between photon B and C, we can acquire the final state, ${|{\phi }_{{\rm{F}}}^{+}\rangle }_{{\rm{ACB}}}$ (three-photon *W* state), as follows:

18 $$\begin{array}{lll}{|{\phi }_{8}^{+}\rangle }_{{\rm{ACB}}} & \mathop{\to }\limits^{{\rm{CPBS}}} & \frac{1}{2}({|R\rangle }_{{\rm{A}}}^{{\rm{a}}}{|R\rangle }_{{\rm{C}}}^{{\rm{c}}}{|R\rangle }_{{\rm{B}}}^{{\rm{b}}}+{|R\rangle }_{{\rm{A}}}^{{\rm{a}}}{|L\rangle }_{{\rm{C}}}^{{\rm{c}}}{|L\rangle }_{{\rm{B}}}^{{\rm{b}}}+\sqrt{2}{|L\rangle }_{{\rm{A}}}^{{\rm{a}}}{|L\rangle }_{{\rm{C}}}^{{\rm{c}}}{|R\rangle }_{{\rm{B}}}^{{\rm{b}}})\\  & \mathop{\to }\limits^{\mathrm{feed}-\mathrm{forward}} & \frac{1}{2}({|R\rangle }_{{\rm{A}}}^{{\rm{a}}}{|L\rangle }_{{\rm{C}}}^{{\rm{c}}}{|R\rangle }_{{\rm{B}}}^{{\rm{b}}}+{|R\rangle }_{{\rm{A}}}^{{\rm{a}}}{|R\rangle }_{{\rm{C}}}^{{\rm{c}}}{|L\rangle }_{{\rm{B}}}^{{\rm{b}}}+\sqrt{2}{|L\rangle }_{{\rm{A}}}^{{\rm{a}}}{|R\rangle }_{{\rm{C}}}^{{\rm{c}}}{|R\rangle }_{{\rm{B}}}^{{\rm{b}}})\\  & \mathop{\to }\limits^{{\rm{crossing}}\,{\rm{path}}} & {|{\phi }_{{\rm{F}}}^{+}\rangle }_{{\rm{ABC}}}=\frac{1}{2}({|R\rangle }_{{\rm{A}}}^{{\rm{a}}}{|R\rangle }_{{\rm{B}}}^{{\rm{b}}}{|L\rangle }_{{\rm{C}}}^{{\rm{c}}}+{|R\rangle }_{{\rm{A}}}^{{\rm{a}}}{|L\rangle }_{{\rm{B}}}^{{\rm{b}}}{|R\rangle }_{{\rm{C}}}^{{\rm{c}}}+\sqrt{2}{|L\rangle }_{{\rm{A}}}^{{\rm{a}}}{|R\rangle }_{{\rm{B}}}^{{\rm{b}}}{|R\rangle }_{{\rm{C}}}^{{\rm{c}}}),\end{array}$$

Also, if we suppose that the result of parity gate using the QD-cavity system is in the state ${|{-}_{e}\rangle }_{1}$, then we can obtain the output state ${|{\phi }_{8}^{-}\rangle }_{{\rm{ACB}}}$ of CNOT gate as ${|{\phi }_{8}^{-}\rangle }_{{\rm{ACB}}}=(-{|R\rangle }_{{\rm{A}}}^{1}{|L\rangle }_{{\rm{C}}}^{{\rm{c}}}{|R\rangle }_{{\rm{B}}}^{{\rm{b}}}+{|R\rangle }_{{\rm{A}}}^{1}{|R\rangle }_{{\rm{C}}}^{{\rm{c}}}{|L\rangle }_{{\rm{B}}}^{{\rm{b}}}-\sqrt{2}{|L\rangle }_{{\rm{A}}}^{2}{|R\rangle }_{{\rm{C}}}^{{\rm{c}}}{|R\rangle }_{{\rm{B}}}^{{\rm{b}}})/2$. To generate *W* state as shown in Eq. 18, we should apply PF (phase flipper) to a photon B by feed-forward (red-dotted box in Fig. 3).

So far, we have designed an optical scheme to generate three-photon *W* state with robust entanglement against loss of one photon using XKNLs and QD-cavity system. In the next section, we will analyze the efficiency and performance of nonlinearly optical gates for its implementation in practice.

## Analysis of Performance and Efficiency in Nonlinearly Optical Gates Using XKNLs and QD-Cavity System

### Controlled-path and merging-path gates under decoherence

**Figure 7:**
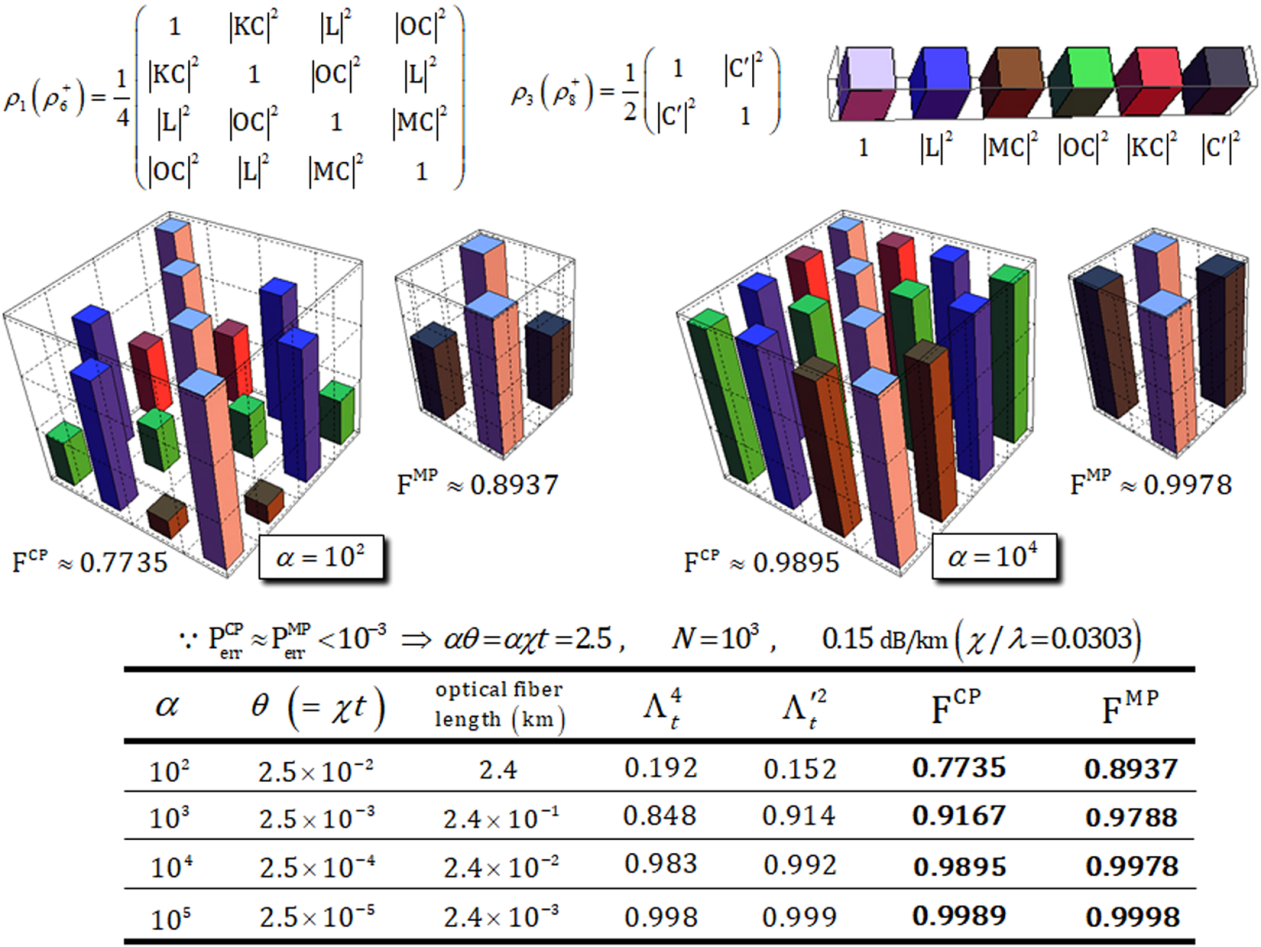
Big (small) diagram represents the density matrix, *ρ*_1_ or ${\rho }_{6}^{+}$ (*ρ*_3_ or ${\rho }_{8}^{+}$) of output state, which applied to the process model in Eq. 20, after controlled-path (merging-path) gate. These diagrams obviously show that values of coherent parameters approach 1 and fidelities F^CP^ (of controlled-path gate) and F^CP^ (of merging-path gate) are increased when using strong (large amplitude) coherent state for *αθ* = *αχt* = 2.5 and *N* = 10^3^ with signal loss of 0.15 dB/km (*χ*/*λ* = 0.0303) in optical fiber^71^. In the Table, fidelities and the rate of ${{\rm{\Lambda }}}_{t}^{4}$ (${{\rm{\Lambda }}^{\prime} }_{t}^{2}$), of the remaining photon in probe beams of controlled-path gate (merging-path gate) are calculated in accordance with differences in amplitude, *α*, of coherent state. In addition, when *αθ* = *αχt* = 2.5 is fixed for ${{\rm{P}}}_{{\rm{err}}}^{{\rm{CP}}}\approx {{\rm{P}}}_{{\rm{err}}}^{{\rm{MP}}} < {10}^{-3}$, the magnitude of conditional phase shift, *θ*, and length of optical fiber needed are also listed in Table.

In optical fibers (practice), the decoherence effect inevitably results in photon loss and dephasing of coherent parameters^41,42,46,55–57^ when nonlinearly optical gates (controlled-path and merging-path gates) are realized in Kerr medium. This influence (decoherence)^41,42,46^ on nonlinearly optical gates using XKNLs can be analytically represented by the solution of master equation^69^ to describe an open quantum system, as follows:

19 $$\frac{\partial \rho (t)}{\partial t}=-\,\frac{i}{\hslash }[H,\rho ]+\lambda ({a}\rho {{a}}^{+}+\frac{1}{2}({{a}}^{+}{a}\rho +\rho {{a}}^{+}{a})),\,\because \hat{J}\rho =\lambda {a}\rho {{a}}^{+},\,\hat{L}\rho =-\,\frac{\lambda }{2}({{a}}^{+}{a}\rho +\rho {{a}}^{+}{a})$$

where *λ*, *t* (=*θ*/*χ*), and *a*^+^ (*a*) are energy decay rate, interaction time, and creation (annihilation) operator, respectively. The solution of master equation can be calculated as $\rho (t)=\exp [(\hat{J}+\hat{L})t]\rho (0)$^58^. Thus, we can introduce the process model^41,42,46^ of decoherence effect (photon loss and dephasing), ${\hat{D}}_{t}$, and conditional phase shift (θ) by XKNLs, ${\hat{X}}_{t}$, from the solution of master equation in signal-probe (photon-coherent state) system, as follows:

20 $${({\tilde{D}}_{{\rm{\Delta }}t}{\tilde{X}}_{{\rm{\Delta }}t})}^{N}|H\rangle \langle V|\otimes |\alpha \rangle \langle \alpha |=\exp [-{\alpha }^{2}(1-{e}^{-\lambda {\rm{\Delta }}t})\sum _{n=1}^{N}{e}^{-\lambda {\rm{\Delta }}t(n-1)}(1-{e}^{in{\rm{\Delta }}\theta })]|H\rangle \langle V|\otimes |{{\rm{\Lambda }}}_{t}\alpha {e}^{i\theta }\rangle \langle {{\rm{\Lambda }}}_{t}\alpha |,$$

where ${\hat{X}}_{t}|H\rangle |\alpha \rangle \Rightarrow |H\rangle |\alpha {e}^{i\theta }\rangle$ and ${\hat{D}}_{t}{\hat{X}}_{t}={({\hat{D}}_{{\rm{\Delta }}t}{\hat{X}}_{{\rm{\Delta }}t})}^{N}$ for the divided interaction time Δ*t* (=*t*/*N*) with *θ* = *χt* = *χN*Δ*t* = *N*Δ*θ*. Λ_*t*_ = *e*^−*λt*/2^ is the rate of remaining photon in probe beam, due to photon loss in Kerr medium^41,42,46^. To analyze the process model, we take pure silica core fibers^70,71^, that require a length of about 3000 km for conditional phase shift, *θ* = *π*, by XKNL^41,42,46^ with signal loss of *χ*/*λ* = 0.0303 (0.15 dB/km), to experimentally realize controlled-path and merging-path gates, in practice. By the process model (Eq. 20) considering the decoherence effect on nonlinearly optical gates, output states (Eqs 8, 10, 15 and 17) can be evolved to mixed states, as follows:

21 $${\rho }_{1}={\rho }_{6}^{+}=\frac{1}{4}(\begin{array}{cccc}1 & {|{\rm{K}}{\rm{C}}|}^{2} & {|{\rm{L}}|}^{2} & {|{\rm{O}}{\rm{C}}|}^{2}\\ {|{\rm{K}}{\rm{C}}|}^{2} & 1 & {|{\rm{O}}{\rm{C}}|}^{2} & {|{\rm{L}}|}^{2}\\ {|{\rm{L}}|}^{2} & {|{\rm{O}}{\rm{C}}|}^{2} & 1 & {|{\rm{M}}{\rm{C}}|}^{2}\\ {|{\rm{O}}{\rm{C}}|}^{2} & {|{\rm{L}}|}^{2} & {|{\rm{M}}{\rm{C}}|}^{2} & 1\end{array}),\,{\rho }_{3}={\rho }_{8}^{+}=\frac{1}{2}(\begin{array}{cc}1 & {|{{\rm{C}}}^{{\rm{^{\prime} }}}|}^{2}\\ {|{{\rm{C}}}^{{\rm{^{\prime} }}}|}^{2} & 1\end{array}),\,\because \begin{array}{c}{|{\phi }_{1}\rangle }_{{\rm{A}}{\rm{B}}{\rm{C}}\otimes {\rm{P}}}\,({\rm{E}}{\rm{q}}.\,8)\Rightarrow {\rho }_{1}\\ {|{\phi }_{3}\rangle }_{{\rm{A}}{\rm{B}}{\rm{C}}\otimes {\rm{P}}}\,({\rm{E}}{\rm{q}}.\,10)\Rightarrow {\rho }_{3}\\ {|{\phi }_{6}^{+}\rangle }_{{\rm{A}}{\rm{C}}{\rm{B}}\otimes {\rm{P}}}\,({\rm{E}}{\rm{q}}.\,15)\Rightarrow {\rho }_{6}^{+}\\ {|{\phi }_{8}^{+}\rangle }_{{\rm{A}}{\rm{C}}{\rm{B}}\otimes {\rm{P}}}\,({\rm{E}}{\rm{q}}.\,17)\Rightarrow {\rho }_{8}^{+}\end{array}$$

where off-diagonal terms in matrices are called coherent parameters. Also, *ρ*_1_ and ${\rho }_{6}^{+}$ of controlled-path gate (*ρ*_3_ and ${\rho }_{8}^{+}$ of merging-path gate) are identical forms of density matrices. They are defined by different basis sets, as follows:

22 $$\begin{array}{c}{\rho }_{{\rm{1}}}:\{{|R\rangle }_{{\rm{A}}}^{1}{|R\rangle }_{{\rm{B}}}^{3}{|H\rangle }_{{\rm{C}}}^{{\rm{c}}}\otimes {|{{\rm{\Lambda }}}_{t}^{2}\alpha \rangle }_{{\rm{P}}}^{{\rm{u}}}{|0\rangle }_{{\rm{P}}}^{{\rm{d}}},{|L\rangle }_{{\rm{A}}}^{2}{|R\rangle }_{{\rm{B}}}^{4}{|H\rangle }_{{\rm{C}}}^{{\rm{c}}}\otimes {|{{\rm{\Lambda }}}_{t}^{2}\alpha \rangle }_{{\rm{P}}}^{{\rm{u}}}{|0\rangle }_{{\rm{P}}}^{{\rm{d}}},\\ \,\,\,\,{|R\rangle }_{{\rm{A}}}^{1}{|R\rangle }_{{\rm{B}}}^{4}{|H\rangle }_{{\rm{C}}}^{{\rm{c}}}\otimes {|{{\rm{\Lambda }}}_{t}^{2}\alpha \,\cos \,{\rm{\theta }}\rangle }_{{\rm{P}}}^{{\rm{u}}}{|i{{\rm{\Lambda }}}_{t}^{2}\alpha \,\sin \,{\rm{\theta }}\rangle }_{{\rm{P}}}^{{\rm{d}}},\\ \,\,\,\,{|R\rangle }_{{\rm{A}}}^{2}{|R\rangle }_{{\rm{B}}}^{3}{|H\rangle }_{{\rm{C}}}^{{\rm{c}}}\otimes {|{{\rm{\Lambda }}}_{t}^{2}\alpha \,\cos \,{\rm{\theta }}\rangle }_{{\rm{P}}}^{{\rm{u}}}{|\,-\,i{{\rm{\Lambda }}}_{t}^{2}\alpha \,\sin \,{\rm{\theta }}\rangle }_{{\rm{P}}}^{{\rm{d}}}\},\\ {\rho }_{6}^{+}:\{\frac{1}{\sqrt{2}}(\,-\,{|R\rangle }_{{\rm{A}}}^{1}{|L\rangle }_{{\rm{C}}}^{{\rm{5}}}{|R\rangle }_{{\rm{B}}}^{{\rm{b}}}-{|R\rangle }_{{\rm{A}}}^{1}{|R\rangle }_{{\rm{C}}}^{{\rm{5}}}{|L\rangle }_{{\rm{B}}}^{{\rm{b}}})\otimes {|{{\rm{\Lambda }}}_{t}^{2}\alpha \rangle }_{{\rm{P}}}^{{\rm{u}}}{|0\rangle }_{{\rm{P}}}^{{\rm{d}}},{|L\rangle }_{{\rm{A}}}^{2}{|L\rangle }_{{\rm{C}}}^{{\rm{6}}}{|R\rangle }_{{\rm{B}}}^{{\rm{b}}}\otimes {|{{\rm{\Lambda }}}_{t}^{2}\alpha \rangle }_{{\rm{P}}}^{{\rm{u}}}{|0\rangle }_{{\rm{P}}}^{{\rm{d}}},\\ \,\,\,\,\frac{1}{\sqrt{2}}(\,-\,{|R\rangle }_{{\rm{A}}}^{1}{|L\rangle }_{{\rm{C}}}^{{\rm{6}}}{|R\rangle }_{{\rm{B}}}^{{\rm{b}}}-{|R\rangle }_{{\rm{A}}}^{1}{|R\rangle }_{{\rm{C}}}^{{\rm{6}}}{|L\rangle }_{{\rm{B}}}^{{\rm{b}}})\otimes {|{{\rm{\Lambda }}}_{t}^{2}\alpha \,\cos \,{\rm{\theta }}\rangle }_{{\rm{P}}}^{{\rm{u}}}{|i{{\rm{\Lambda }}}_{t}^{2}\alpha \,\sin \,{\rm{\theta }}\rangle }_{{\rm{P}}}^{{\rm{d}}},\\ \,\,\,\,{|L\rangle }_{{\rm{A}}}^{2}{|L\rangle }_{{\rm{C}}}^{{\rm{5}}}{|R\rangle }_{{\rm{B}}}^{{\rm{b}}}\otimes {|{{\rm{\Lambda }}}_{t}^{2}\alpha \,\cos \,{\rm{\theta }}\rangle }_{{\rm{P}}}^{{\rm{u}}}{|\,-\,i{{\rm{\Lambda }}}_{t}^{2}\alpha \,\sin \,{\rm{\theta }}\rangle }_{{\rm{P}}}^{{\rm{d}}}\},\\ {\rho }_{3}:\{\frac{1}{2}(\,-\,{|R\rangle }_{{\rm{A}}}^{1}{|R\rangle }_{{\rm{B}}}^{{\rm{3}}}-{|R\rangle }_{{\rm{A}}}^{1}{|L\rangle }_{{\rm{B}}}^{{\rm{3}}}+\sqrt{2}{|L\rangle }_{{\rm{A}}}^{2}{|R\rangle }_{{\rm{B}}}^{{\rm{3}}}){|H\rangle }_{{\rm{C}}}^{{\rm{c}}}\otimes {|{{\rm{\Lambda }}^{\prime} }_{t}\beta \rangle }_{{\rm{P}}}^{{\rm{u}}}{|0\rangle }_{{\rm{P}}}^{{\rm{d}}},\\ \,\,\,\,\frac{1}{2}({|R\rangle }_{{\rm{A}}}^{1}{|R\rangle }_{{\rm{B}}}^{{\rm{4}}}+{|R\rangle }_{{\rm{A}}}^{1}{|L\rangle }_{{\rm{B}}}^{{\rm{4}}}+\sqrt{2}{|L\rangle }_{{\rm{A}}}^{2}{|R\rangle }_{{\rm{B}}}^{{\rm{4}}}){|H\rangle }_{{\rm{C}}}^{{\rm{c}}}\otimes {|{{\rm{\Lambda }}^{\prime} }_{t}\beta \,\cos \,{\rm{\theta }}\rangle }_{{\rm{P}}}^{{\rm{u}}}{|i{{\rm{\Lambda }}^{\prime} }_{t}\beta \,\sin \,{\rm{\theta }}\rangle }_{{\rm{P}}}^{{\rm{d}}}\},\\ {\rho }_{8}^{+}:\{\frac{1}{2}({|R\rangle }_{{\rm{A}}}^{1}{|R\rangle }_{{\rm{C}}}^{{\rm{5}}}{|R\rangle }_{{\rm{B}}}^{{\rm{b}}}+{|R\rangle }_{{\rm{A}}}^{1}{|L\rangle }_{{\rm{C}}}^{{\rm{5}}}{|L\rangle }_{{\rm{B}}}^{{\rm{b}}}+\sqrt{2}{|L\rangle }_{{\rm{A}}}^{2}{|L\rangle }_{{\rm{C}}}^{{\rm{5}}}{|R\rangle }_{{\rm{B}}}^{{\rm{b}}}){|H\rangle }_{{\rm{C}}}^{{\rm{c}}}\otimes {|{{\rm{\Lambda }}^{\prime} }_{t}\beta \rangle }_{{\rm{P}}}^{{\rm{u}}}{|0\rangle }_{{\rm{P}}}^{{\rm{d}}},\\ \,\,\,\,\frac{1}{2}(\,-\,{|R\rangle }_{{\rm{A}}}^{1}{|R\rangle }_{{\rm{C}}}^{{\rm{6}}}{|R\rangle }_{{\rm{B}}}^{{\rm{b}}}-{|R\rangle }_{{\rm{A}}}^{1}{|L\rangle }_{{\rm{C}}}^{{\rm{6}}}{|L\rangle }_{{\rm{B}}}^{{\rm{b}}}+\sqrt{2}{|L\rangle }_{{\rm{A}}}^{2}{|L\rangle }_{{\rm{C}}}^{{\rm{6}}}{|R\rangle }_{{\rm{B}}}^{{\rm{b}}}){|H\rangle }_{{\rm{C}}}^{{\rm{c}}}\otimes {|{{\rm{\Lambda }}^{\prime} }_{t}\beta \,\cos \,{\rm{\theta }}\rangle }_{{\rm{P}}}^{{\rm{u}}}{|i{{\rm{\Lambda }}^{\prime} }_{t}\beta \,\sin \,{\rm{\theta }}\rangle }_{{\rm{P}}}^{{\rm{d}}}\},\end{array}$$

where Λ_*t*_ = *e*^−*λt*/2^ is the rate of remaining photon through controlled-path gate and Λ′_*t*_ = *e*^−*λ*′*t*/2^ is the rate of remaining photon through merging-path gate, assuming the probe beam, as ${|\alpha \,\cos \,{\rm{\theta }}\rangle }_{{\rm{P}}}^{{\rm{u}}}\equiv {|\beta \rangle }_{{\rm{P}}}^{{\rm{u}}}$, of merging-path gate after measurement in the controlled-path gate as described in Sec. 3. Then we can calculate coherent parameters of (C, M, L, O, K, and C′) in Eq. 21 from the process model (Eq. 20), as follows:

23 $$\begin{array}{c}{\rm{C}}=\exp [\,-\,\frac{{\alpha }^{2}}{2}(1-{e}^{-\lambda {\rm{\Delta }}t})\sum _{n=1}^{N}{e}^{-\lambda {\rm{\Delta }}t(n-1)}(1-{e}^{in{\rm{\Delta }}\theta })],\\ {\rm{M}}=\exp [\,-\,\frac{{\alpha }^{2}}{2}{e}^{-\lambda t}(1-{e}^{-\lambda {\rm{\Delta }}t})\sum _{n=1}^{N}{e}^{-\lambda {\rm{\Delta }}t(n-1)}(1-{e}^{i(n{\rm{\Delta }}\theta +\theta )})],\\ {\rm{L}}=\exp [\,-\,\frac{{\alpha }^{2}}{2}{e}^{-\lambda t}(1-{e}^{-\lambda {\rm{\Delta }}t})\sum _{n=1}^{N}{e}^{-\lambda {\rm{\Delta }}t(n-1)}(1-{e}^{in{\rm{\Delta }}\theta })],\\ {\rm{O}}=\exp [\,-\,\frac{{\alpha }^{2}}{2}{e}^{-\lambda t}(1-{e}^{-\lambda {\rm{\Delta }}t})(1-{e}^{i\theta })\sum _{n=1}^{N}{e}^{-\lambda {\rm{\Delta }}t(n-1)}],\\ {\rm{K}}=\exp [\,-\,\frac{{\alpha }^{2}}{2}{e}^{-\lambda t}(1-{e}^{-\lambda {\rm{\Delta }}t})\sum _{n=1}^{N}{e}^{-\lambda {\rm{\Delta }}t(n-1)}(1-{e}^{-i(n{\rm{\Delta }}\theta -\theta )})],\\ {{\rm{C}}}^{{\rm{^{\prime} }}}=\exp [\,-\,\frac{{\beta }^{2}}{2}(1-{e}^{-{\lambda }^{{\rm{^{\prime} }}}{\rm{\Delta }}t})\sum _{n=1}^{N}{e}^{-{\lambda }^{{\rm{^{\prime} }}}{\rm{\Delta }}t(n-1)}(1-{e}^{in{\rm{\Delta }}\theta })],\end{array}$$

where ${\hat{D}}_{t}{\hat{X}}_{t}={({\hat{D}}_{{\rm{\Delta }}t}{\hat{X}}_{{\rm{\Delta }}t})}^{N}$ and *θ* = *χt* = *χN*Δ*t* = *N*Δ*θ* for divided interaction time Δ*t* (=*t*/*N*) with *α* ∈ **R**. We can quantify the influence of decoherence effect (photon loss and dephasing) to calculate fidelities of output states of nonlinearly optical gates for the reliable performance and efficiency of controlled-path and merging-path gates in our scheme (generation of *W* state). To analyze the influence of the decoherence effect, we take to fix the parameters *αθ* = *αχt* = 2.5 (for ${{\rm{P}}}_{{\rm{err}}}^{{\rm{CP}}}\approx {{\rm{P}}}_{{\rm{err}}}^{{\rm{MP}}} < {10}^{-3}$) and *N* = 10^3^ (for a good approximation) with *α* ≫ 10 and *θ* ≪ 0.1, and assume to realize the nonlinearly optical gates in optical fiber^71^ having the signal loss of 0.15 dB/km (*χ*/*λ* = 0.0303). Figure 7 shows high efficiency and reliable performance of controlled-path and merging-path gates under the decoherence effect to acquire high rates of ${{\rm{\Lambda }}}_{t}^{4}$ (of controlled-path gate) and ${{\rm{\Lambda }}^{\prime} }_{t}^{2}$ (of merging-path gate) of the remaining photon with increasing fidelities of F^CP^ (of controlled-path gate) and F^MP^ (of merging-path gate) by using strong amplitude, *α* > 10^3^, of coherent state via our analysis from the process model of Eq. 20. Here, rates of the remaining photon and fidelities are given by

24 $$\begin{array}{ccc}{{\rm{c}}{\rm{o}}{\rm{n}}{\rm{t}}{\rm{r}}{\rm{o}}{\rm{l}}{\rm{l}}{\rm{e}}{\rm{d}}-{\rm{p}}{\rm{a}}{\rm{t}}{\rm{h}}:{\rm{\Lambda }}}_{t} & = & {e}^{-\lambda t/2},\\ {{\rm{F}}}^{{\rm{C}}{\rm{P}}} & \equiv  & |\sqrt{\langle {\phi }_{1}|{\rho }_{1}|{\phi }_{1}\rangle }|=|\sqrt{\langle {\phi }_{6}|{\rho }_{6}^{+}|{\phi }_{6}\rangle }|\\  & = & \frac{1}{2}|\sqrt{1+{|{\rm{L}}|}^{2}+{|{\rm{O}}{\rm{C}}|}^{2}+({|{\rm{K}}{\rm{C}}|}^{2}+{|{\rm{M}}{\rm{C}}|}^{2})/2}|,\\ {{\rm{m}}{\rm{e}}{\rm{r}}{\rm{g}}{\rm{i}}{\rm{n}}{\rm{g}}-{\rm{p}}{\rm{a}}{\rm{t}}{\rm{h}}:{{\rm{\Lambda }}}^{{\rm{^{\prime} }}}}_{t} & = & {e}^{-{\lambda }^{{\rm{^{\prime} }}}t/2},\\ {{\rm{F}}}^{{\rm{M}}{\rm{P}}} & \equiv  & |\sqrt{\langle {\phi }_{3}|{\rho }_{3}|{\phi }_{3}\rangle }|=|\sqrt{\langle {\phi }_{8}|{\rho }_{8}^{+}|{\phi }_{8}\rangle }|\\  & = & \frac{1}{\sqrt{2}}|\sqrt{1+{|{\rm{C}}|}^{2}}|,\end{array}$$

where fixed parameters are *αθ* = *αχt* = 2.5 (${{\rm{P}}}_{{\rm{err}}}^{{\rm{CP}}}\approx {{\rm{P}}}_{{\rm{err}}}^{{\rm{MP}}} < {10}^{-3}$) and *N* = 10^3^ with signal loss as 0.15 dB/km (*χ*/*λ* = 0.0303) in optical fiber. Thus, we can take parameters (including conditions of *αθ* = *αχt* = 2.5, *N* = 10^3^, and *χ*/*λ* = 0.0303) into Eq. 23 (coherent parameters) and 24 to analyze the influence (photon loss and dephasing) of decoherence effect, such as *θ* = 2.5/*α* (Δ*θ* = 2.5/10^3^ · *α*), *λt* = 2.5/0.0303 · *α* (*λ*Δ*t* = 2.5/0.0303 · 10^3^ · *α*), and *λ*′*t* = 2.5/0.0303 · *β* (*λ*′Δ*t* = 2.5/0.0303 · 10^3^ · *β*) for $\beta =\alpha \,\cos \,{\rm{\theta }}$. When strong amplitude of coherent state is employed in nonlinearly optical gates, we can confirm that values of coherent parameters in density matrices (*ρ*_1_, ${\rho }_{6}^{+}$, *ρ*_3_, and ${\rho }_{8}^{+}$) increase as shown in diagrams of Fig. 7. This means that we can obtain high fidelities (F^CP^, F^MP^ → 1), and also maintain output states into pure states against decoherence effect for reliable performance. Moreover, as shown in the Table of Fig. 7, after interactions of XKNLs, rates of remaining photon approach 1 (${{\rm{\Lambda }}}_{t}^{4},\,{{\rm{\Lambda }}^{\prime} }_{t}^{2}\to 1$: decreasing rate of loss) if amplitude of coherent state for *αθ* = *αχt* = 2.5 and *N* = 10^3^ is increased with signal loss of 0.15 dB/km (*χ*/*λ* = 0.0303). Furthermore, as listed in Table, if we increase the amplitude of coherent state, the magnitude of conditional phase shift is smaller (weak XKNL) and also the length of optical fiber is shorter (a short optical fiber length for XKNL) to drive conditional operation in Kerr medium (i.e., if *α* = 10^5^, needed conditions as *θ* = 2.5 × 10^−5^ and 0.0024 km). Namely, his result also demonstrates the feasibility of experimental implementation of nonlinearly optical gates by using weak XKNL and short length of optical fiber. As a result, we can obtain high efficiency and reliable performance (high rates of remaining photon and high fidelities, as described in Fig. 7) of nonlinearly optical gates, in practice, to increase the amplitude of coherent state (strong probe beam) for *αθ* = *αχt* = 2.5 and *N* = 10^3^ in optical fiber having signal loss of 0.15 dB/km (*χ*/*λ* = 0.0303) under the decoherence effect.

### Parity gate using the QD-cavity system with noise

For reliable performance of the QD-cavity system that can realize nonlinearly optical gate (parity gate in Sec. 3) between photons and an electron, we should consider reflection coefficient *R*(*ω*) (hot cavity: *R*_h_(*ω*) and cold cavity: *R*_0_(*ω*)) with the noise *N*(*ω*) and leakage *S*(*ω*) coefficients^4,20^ in practice. For the practical reflection operator, ${\hat{{\rm{R}}}}_{{\rm{P}}}$, of the QD-cavity system, cavity mode operator $\hat{{a}}$ and the dipole operator ${\hat{\sigma }}_{-}$ of X^−^, including noise, sideband leakage, and absorption can be expressed by Heisenberg equation of motion and input-output relations^64^, as follows:

25 $$\begin{array}{ccc}\frac{d\hat{a}}{dt} & = & -[i({\omega }_{c}-\omega )+\frac{\kappa }{2}+\frac{{\kappa }_{s}}{2}]\hat{a}-g{\hat{\sigma }}_{-}-\sqrt{\kappa }{\hat{b}}_{{\rm{i}}{\rm{n}}}-\sqrt{{\kappa }_{s}}{\hat{S}}_{{\rm{i}}{\rm{n}}},\\ \frac{d{\hat{\sigma }}_{-}}{dt} & = & -[i({\omega }_{{{\rm{X}}}^{-}}-\omega )+\frac{\gamma }{2}]{\hat{\sigma }}_{-}-g{\hat{\sigma }}_{Z}\hat{a}+\sqrt{\gamma }{\hat{\sigma }}_{Z}\hat{N},\\ {\hat{b}}_{{\rm{o}}{\rm{u}}{\rm{t}}} & = & {\hat{b}}_{{\rm{i}}{\rm{n}}}+\sqrt{\kappa }\hat{a},\,\,\,\,{\hat{S}}_{{\rm{o}}{\rm{u}}{\rm{t}}}={\hat{S}}_{{\rm{i}}{\rm{n}}}+\sqrt{\kappa }\hat{a},\end{array}$$

where ${\hat{S}}_{{\rm{in}}}({\hat{S}}_{{\rm{out}}})$ is an input (output) field operator from leaky modes due to sideband leakage and absorption, and $\hat{N}$ is vacuum noise operator for ${\hat{\sigma }}_{-}$. From Eq. 25, noise *N*(*ω*) and leakage *S*(*ω*) coefficients^4,20^ are calculated as

26 $$\begin{array}{c}N(\omega )=\frac{\sqrt{\gamma \kappa }g}{[i({\omega }_{c}-\omega )+\gamma /2][i({\omega }_{c}-\omega )+\kappa /2+{\kappa }_{s}/2]+{g}^{2}},\\ S(\omega )=\frac{-\sqrt{{\kappa }_{s}\kappa }[i({\omega }_{c}-\omega )+\gamma /2]}{[i({\omega }_{c}-\omega )+\gamma /2][i({\omega }_{c}-\omega )+\kappa /2+{\kappa }_{s}/2]+{g}^{2}},\end{array}$$

where output field operator ${\hat{{b}}}_{{\rm{out}}}=R(\omega ){\hat{{b}}}_{{\rm{in}}}+S(\omega ){\hat{S}}_{{\rm{in}}}+N(\omega )\hat{N}$ and ground state in QD, $\langle {\hat{\sigma }}_{Z}\rangle \approx -\,1$, with ${\omega }_{c}={\omega }_{{{\rm{X}}}^{-}}$ in weak approximation^64^. Then, we can calculate noise |*n*_h_(*ω*)| and leakage |*s*_h_(*ω*)| rates from coefficients in Eq. 26, in the hot cavity, *g* ≠ 0 (coupled with QD and cavity), as follows:

27 $${N}_{{\rm{h}}}(\omega )=N(\omega )\equiv |{n}_{{\rm{h}}}(\omega )|{e}^{i{\phi }_{{\rm{n}}{\rm{h}}}(\omega )},\,\,{S}_{{\rm{h}}}(\omega )=S(\omega )\equiv |{s}_{{\rm{h}}}(\omega )|{e}^{i{\phi }_{{\rm{s}}{\rm{h}}}(\omega )},$$

where ${\phi }_{{\rm{nh}}}(\omega )\equiv {\rm{\arg }}({N}_{{\rm{h}}}(\omega ))$ and ${\phi }_{{\rm{sh}}}(\omega )\equiv \text{arg}({S}_{{\rm{h}}}(\omega ))$ are phase shifts by noise and leakage. Also, in the cold cavity, *g* = 0 (uncoupled with QD and cavity), noise |*n*_0_(*ω*)| and leakage |*s*_0_(*ω*)| rates are given by

28 $${N}_{0}(\omega )\equiv |{n}_{0}(\omega )|{e}^{i{\phi }_{{\rm{n}}0}(\omega )}=0,\,\,{S}_{0}(\omega )\equiv |{s}_{0}(\omega )|{e}^{i{\phi }_{{\rm{s}}0}(\omega )}=\frac{-\sqrt{{\kappa }_{s}\kappa }}{i({\omega }_{c}-\omega )+\kappa /2+{\kappa }_{s}/2},$$

where ${\phi }_{{\rm{n0}}}(\omega )\equiv {\rm{\arg }}({N}_{{\rm{0}}}(\omega ))$ and ${\phi }_{{\rm{s0}}}(\omega )\equiv {\rm{\arg }}({S}_{{\rm{0}}}(\omega ))$ are phase shifts by noise and leakage. In Sec. 2, reflection coefficients, *R*_h_(*ω*) = *R*(*ω*) and *R*_0_(*ω*), are shown in Eqs 4 and 5, respectively. Therefore, we can establish practical reflection operator ${\hat{{\rm{R}}}}_{{\rm{P}}}$, which can describe the interaction between a photon and an electron in QD of the reflected photon and the confined QD in cavity after interaction in the QD-cavity system with practical conditions, as follows:

29 $$\begin{array}{c}{\hat{{\rm{R}}}}_{{\rm{P}}}=({R}_{{\rm{h}}}+{N}_{{\rm{h}}}+{S}_{{\rm{h}}})(|R\rangle \langle R|\otimes |\downarrow \rangle \langle \downarrow |+|L\rangle \langle L|\otimes |\uparrow \rangle \langle \uparrow |)\\ \,\,+({R}_{{\rm{0}}}+{S}_{{\rm{0}}})(|R\rangle \langle R|\otimes |\uparrow \rangle \langle \uparrow |+|L\rangle \langle L|\otimes |\downarrow \rangle \langle \downarrow |),\end{array}$$

where *R*_h_(*ω*), *N*_h_(*ω*), and *S*_h_(*ω*) are reflection, noise, and leakage coefficients, respectively, in Eqs 4 and 27. *R*_0_(*ω*), *N*_0_(*ω*), and *S*_0_(*ω*) are reflection, noise, and leakage coefficients, respectively, in Eqs 5 and 28. Compared to reflection operator $\hat{{\rm{R}}}$ (Eq. 6) omitting vacuum noise $\hat{N}$ and leaky modes $\hat{S}$, we can analyze effects of noise and leakage and the performance of the interaction in the QD-cavity system via practical reflection operator ${\hat{{\rm{R}}}}_{{\rm{P}}}$. In our scheme (generation of *W* state), the parity gate, in Sec. 3, using the QD-cavity system should be performed to acquire the output state as

30 $$|R\rangle |\uparrow \rangle \to -\,i|R\rangle |\uparrow \rangle ,\,\,|R\rangle |\downarrow \rangle \to |R\rangle |\downarrow \rangle ,\,\,|L\rangle |\uparrow \rangle \to |L\rangle |\uparrow \rangle ,\,\,|L\rangle |\downarrow \rangle \to -i|L\rangle |\downarrow \rangle .$$

In the case of reflection operator $\hat{{\rm{R}}}$ (Eq. 6), this result can be obtained from parameters of *g*/*κ* = 2.4, *κ*_*s*_ ≈ 0, *γ*/*κ* = 0.1, and *ω* − *ω*_*c*_ = *κ*/2. However, in the reflection operator $\hat{{\rm{R}}}$ (Eq. 6), effects of vacuum noise $\hat{N}$ and leaky modes $\hat{S}$ are not taken into account. For practical feasibility to analyze practical reflection operator ${\hat{{\rm{R}}}}_{{\rm{P}}}$ with noise $\hat{N}$ and leakage $\hat{S}$, let us assume that the input state of a photon and an electron spin is $|H\rangle \otimes |{+}_{e}\rangle$. After interaction in the QD-cavity system, we can acquire the ideal output state, $|{\varphi }_{{\rm{Id}}}\rangle$, from Eqs 6 or 30 and the practical output state, $|{\varphi }_{{\rm{\Pr }}}\rangle$, from Eq. 29, as follows:

31 $$\begin{array}{rcl}|{\varphi }_{{\rm{Id}}}\rangle  & = & \frac{1}{\sqrt{2}}[\frac{1}{\sqrt{2}}(|R\rangle |\downarrow \rangle +|L\rangle |\uparrow \rangle )-\frac{i}{\sqrt{2}}(|R\rangle |\uparrow \rangle +|L\rangle |\downarrow \rangle )],\\ |{\varphi }_{{\rm{\Pr }}}\rangle  & = & \frac{1}{\sqrt{{\rm{N}}}}[\frac{({R}_{{\rm{h}}}+{N}_{{\rm{h}}}+{S}_{{\rm{h}}})}{\sqrt{2}}(|R\rangle |\downarrow \rangle +|L\rangle |\uparrow \rangle )+\frac{({R}_{{\rm{0}}}+{S}_{{\rm{0}}})}{\sqrt{2}}(|R\rangle |\uparrow \rangle +|L\rangle |\downarrow \rangle )],\end{array}$$

where N = |*R*_h_ + *N*_h_ + *S*_h_|^2^ + |*R*_0_ + *S*_0_|^2^. Then, we can quantify the affection of noise $\hat{N}$ and leakage $\hat{S}$ in the QD-cavity system by comparing fidelity (F^QD^) between $|{\varphi }_{{\rm{Id}}}\rangle$ (ideal case: no noise and leakage) and $|{\varphi }_{{\rm{\Pr }}}\rangle$ (practical case).

32 $$\begin{array}{ccc}{{\rm{F}}}^{{\rm{Q}}{\rm{D}}} & \equiv  & |\sqrt{\langle {\varphi }_{{\rm{I}}{\rm{d}}}|{\varphi }_{Pr}\rangle \langle {\varphi }_{Pr}|{\varphi }_{{\rm{I}}{\rm{d}}}\rangle }|\\  & = & \frac{1}{\sqrt{2{\rm{N}}}}|\sqrt{[({R}_{{\rm{h}}}+{N}_{{\rm{h}}}+{S}_{{\rm{h}}})+i({R}_{0}+{S}_{0})][({R}_{{\rm{h}}}^{\ast }+{N}_{{\rm{h}}}^{\ast }+{S}_{{\rm{h}}}^{\ast })-i({R}_{0}^{\ast }+{S}_{0}^{\ast })]}|.\end{array}$$

**Figure 8:**
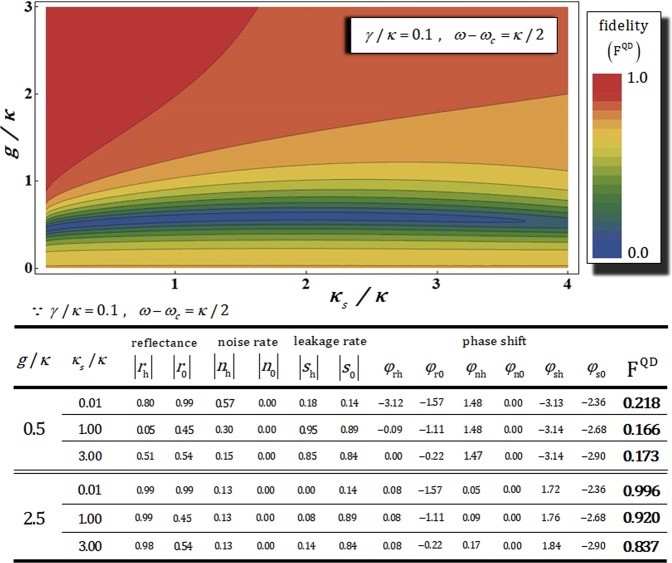
The plot represents the fidelity F^QD^ (the QD-cavity system) of the output state according to side leakage rate *κ*_*s*_/*κ* and coupling strength *g*/*κ* with fixed *γ*/*κ* = 0.1 and *ω* − *ω*_*c*_ = *κ*/2. Values of fidelity, reflectances, noise rates, leakage rates, and phase shifts are listed in the Table, according to differences in *g*/*κ* and *κ*_*s*_/*κ*. This plot and Table obviously show that fidelity approaches 1 when *g*/*κ* (coupling strength between QD and cavity) increases, and *κ*_*s*_/*κ* (side leakage rate of cavity) simultaneously decreases, despite occurred vacuum noise $\hat{N}$ in dipole operation and sideband leakage, absorption $\hat{S}$ in cavity.

Figure 8 shows fidelities F^QD^ of the QD-cavity system and values of reflectances (|*r*_h_| and |*r*_0_|), noise rates (|*n*_h_| and |*n*_0_|), leakage rates (|*s*_h_| and |*s*_0_|), and phase shifts (*ϕ*_rh_, *ϕ*_r0_, *ϕ*_nh_, *ϕ*_n0_, *ϕ*_sh_, and *ϕ*_s0_) for *κ*_*s*_/*κ* and *g*/*κ* with fixed *γ*/*κ* = 0.1 and *ω* − *ω*_*c*_ = *κ*/2. In our analysis^4,20^, when the QD-cavity system has experimental parameters *g* ≫ (*κ*, *γ*) and *κ*_*s*_ ≪ *κ* with small *γ*^47,65–68^ and ${\omega }_{{{\rm{X}}}^{-}}={\omega }_{c}$, the noise rates and the phase shifts (|*n*_h_|, *ϕ*_nh_) and (|*n*_0_|, *ϕ*_n0_), leakage rates and phase shifts (|*s*_h_|, *ϕ*_sh_) and (|*s*_0_|, *ϕ*_s0_) can be ignored, as shown in Fig. 8. For example, as listed in the Table, if experimental parameters are *κ*_*s*_ = 0.01 and *g*/*κ* = 2.5 with *γ*/*κ* = 0.1 and *ω* − *ω*_*c*_ = *κ*/2, we can obtain high fidelity (F^QD^ ~ 0.996), due to |*n*_h_| ≈ |*s*_0_| ≈ 0.14, |*n*_0_| ≈ |*s*_h_| ≈ 0.00, and *ϕ*_nh_ ≈ *ϕ*_n0_ ≈ 0.00, *ϕ*_sh_ ≈ 1.72, *ϕ*_s0_ ≈ −2.36 from the Table. Namely, the affections of vacuum noise $\hat{N}$ on dipole interaction and leaky modes ${\hat{S}}_{{\rm{in}}}$ (sideband leakage and absorption) in cavity mode can be reduced by choosing parameters *g* ≫ (*κ*, *γ*) (strong coupling) and *κ*_*s*_ ≪ *κ* (small side leakage)^4,20^ for reliable interaction, parity operation, of the QD-cavity system. Consequently, we can achieve high efficiency and reliable performance (high fidelity by reducing affection of noise and leakage, as described in Fig. 8) of the QD-cavity system, in practice, to choose strong coupling strength *g* ≫ (*κ*, *γ*) and small side leakage *κ*_*s*_ ≪ *κ* in optical cavity for parity gate in our scheme (generation of *W* state).

## Conclusions

In this paper, we designed an optical scheme to generate three-photon *W* state having robust entanglement against loss of one photon using XKNLs (controlled-Hadamard and -NOT gates) and QD-cavity system (parity gate) and linearly optical devices. To acquire high efficiency, reliable performance, and experimental feasibility of generation of *W* state, we employed two critical components of nonlinearly optical gates as XKNLs and QD-cavity system in our scheme.

In the case of controlled-unitary (Hadamard and NOT) gates using XKNLs, to acquire immunity under the decoherence effect (photon loss and dephasing), we employed qubus beams and PNR measurements with strong (large amplitude) coherent state based on our analysis in Sec. 4. This usage (qubus and PNR) with strong coherent state can prevent the evolvement from pure state to mixed state caused by the decoherence effect^41,42,46^, and also only apply the positive conditional phase shift, θ. Thus, for experimental feasibility, our gates via XKNLs don’t need minus conditional phase shift, which is known challenging task to change the sign of conditional phase shift (−θ → θ)^72^, and large magnitude of conditional phase shift (natural XKNLs are extremely weak, θ ≈ 10^−18^ ^73^) by increasing the amplitude of coherent state (in our analysis, Fig. 7). Moreover, in Sec. 3, the probe beam (coherent state) in controlled-path gate can be recycled for merging-path gate, as ${|\beta \,\cos \,{\rm{\theta }}\rangle }_{{\rm{P}}}^{{\rm{u}}}$, for the efficiency since PNR measurements are applied on the probe beam of path d in controlled-path and merging-path gates. Also, for the sufficient large strength of XKNL, the various experimental technologies in XKNL have been proposed, such as electromagnetically induced transparency (EIT)^74,75^, circuit electromechanics^76^, an artificial atom^77^, and three-dimensional circuit quantum electrodynamic architecture^78^. And to realize the strong phase shift, Friedler *et al*.^79^ showed the large nonlinear interactions between ultraslow-light pulses or two stopped light pulses^80^ in the regime of EIT. For preventing losses of large absorption in single-photon pulse^81^, He *et al*.^75^ showed to employ EIT and long range interaction for using weak XKNL.

From practical reflection operator, ${\hat{{\rm{R}}}}_{{\rm{P}}}$, of the QD-cavity system in Sec. 4, if the coupling strength, *g*/*κ*, is strong as *g* ≫ (*κ*, *γ*) and side leakage rate *κ*_*s*_/*κ* is small as *κ*_*s*_ ≪ *κ* with *γ*/*κ* = 0.1 and *ω* − *ω*_*c*_ = *κ*/2, we can obtain high fidelity (F^QD^ → 1) of the output state by reducing effect of vacuum noise $\hat{N}$ in dipole interaction and leaky modes ${\hat{S}}_{{\rm{in}}}$ (sideband leakage and absorption) in cavity mode. For these requirements of *g* ≫ (*κ*, *γ*) and *κ*_*s*_ ≪ *κ*, by optimizing the etching process (or improving the sample growth)^66^ with *g*/(*κ*_*s*_ + *κ*) ≈ 2.4, side leakage rate, *κ*_*s*_, can be decreased when In_0.6_Ga_0_._4_As (QDs) has *g* ≈ 80 μeV and Q = 40000. Also, a small side leakage rate can be acquired by improving the quality factor to Q = 215000 (*κ* ≈ 6.2 μeV)^82^. Moreover, in a micropillar cavity at d = 1.5 μm for quality factor Q = 8800, the coupling strength can be achieved to have *g*/(*κ*_*s*_ + *κ*) ≈ 0.5^47^. The coupling strength can be experimentally increased to have *g*/(*κ*_*s*_ + *κ*) ≈ 2.4 for Q = 40000^83^. Bayer *et al*.^84^ have also demonstrated that micropillars with d = 1.5 μm and *γ*/*κ* ≈ 1 μeV (the decay rate of X^−^) could be acquired from In_0.6_Ga_0.4_As/GaAs (QDs) with temperature T ≈ 2 K for strong coupling.

Here, we demonstrated that nonlinearly optical (controlled-Hadamard and -NOT) gates using XKNLs should employ a strong coherent state to acquire high efficiency (low error probability) and reliable performance (high fidelity) under the decoherence effect, due to our process model in Sec. 4. In the previous works (controlled-path and merging gates)^44,85–88^, the affection (photon loss and dephasing) of the decoherence effect have been overlooked in practice. In this point of view, we analyzed the decoherence effect through master equation, in Sec. 4, and proposed the method to enhance affection of photon loss and dephasing by utilizing strong coherent state (probe beam). Thus, compared with the previous works^44,85–88^ (including to other schemes^18,21,89,90^ for generation *W* state using XKNLs), our scheme for the generation of *W* state will be more robust against the decoherence effect.

Consequently, we proposed a scheme of deterministic generation of three-photon *W* state using XKNLs (controlled operations) and QD-cavity system (parity operation). Furthermore, through our analysis, we demonstrated the efficiency (with performance) and experimental feasibility of nonlinearly optical gates with strong coherent state (XKNLs) and strong coupling at small side leakage rate (QD-cavity system) for our scheme to generate *W* state.
